# OsNAC103, a NAC Transcription Factor, Positively Regulates Leaf Senescence and Plant Architecture in Rice

**DOI:** 10.1186/s12284-024-00690-3

**Published:** 2024-02-15

**Authors:** Lina Sun, Hanqin Xu, Juan Song, Xiaoying Yang, XinYi Wang, Haiyan Liu, Mengzhen Pang, Youchuan Hu, Qi Yang, Xiaotong Ning, Shanshan Liang, Siju Zhang, Weijiang Luan

**Affiliations:** College of Life Sciences, Tianjin Key Laboratory of Animal and Plant Resistance, Tianjin Normal University, Tianjin, 300387 China

**Keywords:** NAC transcription factor, Leaf senescence, Plant architecture, Rice

## Abstract

Leaf senescence, the last stage of leaf development, is essential for crop yield by promoting nutrition relocation from senescence leaves to new leaves and seeds. NAC (NAM/ATAF1/ATAF2/CUC2) proteins, one of the plant-specific transcription factors, widely distribute in plants and play important roles in plant growth and development. Here, we identified a new NAC member OsNAC103 and found that it plays critical roles in leaf senescence and plant architecture in rice. *OsNAC103* mRNA levels were dramatically induced by leaf senescence as well as different phytohormones such as ABA, MeJA and ACC and abiotic stresses including dark, drought and high salinity. OsNAC103 acts as a transcription factor with nuclear localization signals at the N terminal and a transcriptional activation signal at the C terminal. Overexpression of *OsNAC103* promoted leaf senescence while *osnac103* mutants delayed leaf senescence under natural condition and dark-induced condition, meanwhile, senescence-associated genes (*SAGs*) were up-regulated in *OsNAC103* overexpression (*OsNAC103*-OE) lines, indicating that OsNAC103 positively regulates leaf senescence in rice. Moreover, *OsNAC103*-OE lines exhibited loose plant architecture with larger tiller angles while tiller angles of *osnac103* mutants decreased during the vegetative and reproductive growth stages due to the response of shoot gravitropism, suggesting that OsNAC103 can regulate the plant architecture in rice. Taken together, our results reveal that OsNAC103 plays crucial roles in the regulation of leaf senescence and plant architecture in rice.

**Supplementary Information:**

The online version contains supplementary material available at 10.1186/s12284-024-00690-3.

## Introduction

Leaf senescence is the last stage of leaf development and an age-dependent degeneration process, which is controlled by endogenous genes in plants and environmental factors. In this process, the color of leaves become yellow and wilting due to chlorophyll (Chl) degradation and the breakdown of chloroplasts, as well as the degradation of nucleic acid, protein and lipid. Meanwhile, the degenerated products in senescent leaf are relocated as nutrients to reproductive or younger organs to reuse (Lim et al. 2007; Taylor et al. 2010). Several stay green mutants were characterized and revealed to involve in Chl degradation and the breakdown of chloroplasts in rice. *sgr*, *nyc1*, *nol* and *nyc3* are non-functional stay-green mutants. *SGR* (*STAY-GREEN*) encodes a conserved chloroplast protein which can recruit Chl degradation enzymes to promote Chl breakdown (Jiang et al. 2007; Park et al. 2007; Sakuraba et al. 2012). *NYC1* (*NON-YELLOW COLORING1*) and *NOL* (*NYC1-LIKE*) encode Chl *b* reductase to catalyze the conversion of Chl *b* to HMChl *a* (7-hydroxymethyl chlorophyll *a*) (Kusaba et al. 2007; Sato et al. 2009; Shimoda et al. 2012), and HMChl *a* can be converted to Chl *a* under the catalyzation of HMChl *a* reductase (Meguro et al. 2011; Piao et al. 2017). Overexpression of *NYC1* or *NOL* can promote Chl degradation to cause leaf yellowish phenotype. *NYC3* (*NON-YELLOW COLORING3*) encodes pheophytin pheophorbide hydrolase (PPH) and can remove phytol residues from pheophytin *a* (phein *a*) to generate pheophorbide *a* (pheide *a*) (Morita et al. 2009; Schelbert et al. 2009). Recently revealed *oscoi1b* knockout mutant is a functional stay-green mutant which remains a high photosynthetic capacity during natural senescence (Lee et al. 2015), and senescence-associated genes (*SAGs*) and ethylene signaling-associated genes were down-regulated in *oscoi1b* mutant, suggesting that the JA receptor OsCOI1b regulates leaf senescence by the crosstalk of JA signaling and ethylene signaling.

The process of leaf senescence is precisely regulated directly or indirectly by internal signals such as developmental age, hormone level (Hensel et al. 1993; Koyama 2018) and external signals including extreme temperature, drought, injury, nutrient deficiency, pathogen infection, UV-B radiation, ozone oxidative stress (Lim et al. 2007; Guo and Gan 2012; Gregersen et al. 2013), while environmental stresses are generally integrated into the regulation of senescence by altering hormone levels in plants. In complex regulatory network during leaf senescence, the transcription factors (TFs) play an important role, for example, several NAC members have been revealed to serve crucial roles. The *NAC* gene family is one of the largest plant-specific TFs, with at least 151 members in rice (Nuruzzaman et al. 2010), and can participate in the regulatory network of leaf senescence. NAC proteins have a highly conserved NAC domain with about 150–160 amino acids (aa) and a highly variable C-terminal which has been identified as transcription regulatory domain (TRD) (Souer et al. 1996; Aida et al. 1997; Kikuchi et al. 2000; Hegedus et al. 2003). In addition, NAC domain of NACs can be divided into A-E five highly conserved regions, and nuclear localization signal and DNA binding domains usually locate in C, D and E regions (Kikuchi et al. 2000; Duval et al. 2002). Genome-wide analysis and microarray analysis in rice and *Arabidopsis* respectively revealed that many NAC TFs were up-regulated in senescent leaf (Nuruzzaman et al. 2010; Breeze et al. 2011). There are at least 117 *NAC* genes in the *Arabidopsis* genome (Nuruzzaman et al. 2010), about 20 of which have been identified to positively or negatively regulate leaf senescence (Cao et al. 2023). However, to date only a few senescence-associated *NAC* genes were functionally characterized in rice.

NACs can regulate leaf senescence through changing Chl catabolic genes and other *SAGs* expression directly or indirectly. For example, ANAC046 in *Arabidopsis*, OsNAC2, OsY37/ONAC011 and OsNAP in rice positively regulate the expression of Chl catabolic genes including *NOL*, *SGR* and *PAO* through directly binding to their promoter regions, promoting Chl degradation, decreasing Chl content and finally leading to leaf senescence phenotype (Liang et al. 2014; Oda-Yamamizo et al. 2016; Mao et al. 2017; El Mannai et al. 2017). ONAC106 and OsNAC109 in rice negatively regulate the expression of Chl catabolic genes such as *NYC* and *SGR*, leading to the inhibition of Chl degradation and the delayed leaf senescence phenotype (Sakuraba et al. 2015; Li et al. 2021).

In addition, NACs can regulate leaf senescence by correlating phytohormones and stresses. ANAC019, ANAC055 and ANAC072 positively regulate ABA-induced leaf senescence while ANAC083/VNI2 negatively regulate ABA-induced leaf senescence in *Arabidopsis* (Yang et al. 2011; Takasaki et al. 2015). ANAC019, ANAC055 and ANAC072 also interact with MYCs to mediate JA-induced Chl degradation (Zhu et al. 2015). OsNAC2, ONAC096 and ONAC054 in rice were revealed to involve in ABA-induced leaf senescence (Mao et al. 2017; Kang et al. 2019; Sakuraba et al. 2020). Ectopic expression of *OsNAC2* leads to an increase of ABA levels via directly up-regulating expression of ABA biosynthetic genes *OsNCED3* and *OsZEP1* as well as down-regulating expression of ABA catabolic gene *OsABA8ox1* during leaf senescence (Mao et al. 2017). *ONAC096* up-regulates the expression levels of *OsABI5* and *OsEEL* to promote leaf senescence (Kang et al. 2019). ONAC054 directly binds the promoters of *OsABI5* and *OsNYC1* to regulate leaf senescence in rice (Sakuraba et al. 2020). AtNAP in *Arabidopsis* interacts with SAG113 to regulate leaf senescence through ABA signal pathway (Guo and Gan 2006; Zhang and Gan 2012). *SAG113*, encodes protein phosphatase type 2C (PP2C), is a key gene associated with stomatal closure in ABA signal pathway (Park et al. 2009; Zhang and Gan 2012; Zhao et al. 2017). ABA-AtNAP-SAG113 module regulate leaf senescence through the inhibition of stomatal closure and acceleration of water loss in plants (Zhang and Gan 2012). *OsPP2C06*, *OsPP2C09* and *OsPP2C68*, homologues of *SAG113* in rice, were up-regulated in *OsNAP* overexpressing transgenic plants to result in the reduction of water loss (Chen et al. 2014). Furthermore, ANAC017, ANAC042, ANAC075, ANAC082 and ANAC090 negatively regulate leaf senescence in *Arabidopsis* through reactive oxygen species (ROS) response, phytohormones and/or abiotic stresses (Wu et al. 2012; Kim et al. 2018; Kan et al. 2021), while ANAC032, ANAC053/NTL4, ANAC087, ANAC092/ORE1/AtNAC2 and its paralog ANAC059/ORS1 positively regulate leaf senescence through ROS response (Aeong et al. 1997; Woo et al. 2004; Balazadeh et al. 2011; Lee et al. 2012; Mahmood et al. 2016; Chen et al. 2023).

In addition to the function in the regulation of leaf senescence, NAC TF members play an important role in the plant architecture. Overexpression of *ONAC106* increases the tiller angle (Sakuraba et al. 2015). *onac054*/*rim1* mutant exhibited dwarf phenotype, suggesting that ONAC054/RIM1 can regulate the plant architecture in rice (Yoshii et al. 2010; Sakuraba et al. 2020). Tiller angle and tiller number, influencing the planting density, light interception, photosynthetic efficiency, disease resistance and grain yield, are important agronomic traits for improving optimal plant architecture in rice (Wang and Li 2008; Jiao et al. 2010; Zhu et al. 2020). Tiller angle of rice is closely related to the shoot gravity response (Wang et al. 2022). It has been shown that asymmetric auxin distribution plays a central role in the gravitropic response. *OsLAZY1* (*OsLA1*) negatively regulates shoot gravity response through the regulation of polar auxin transport (PAT) (Li et al. 2007), which the mutation of *OsLA1* leads to a tiller-spreading phenotype in rice. *PIN-FORMED 1* (*PIN1*) and *PIN2* act as the downstream of *OsLA1* to regulate auxin transport to control rice tiller angle (Xu et al. 2005; Chen et al. 2012; Zhu et al. 2020). Recently identified transcription factor OsbZIP49 affects tiller angle by regulating the local auxin homeostasis (Ding et al. 2021). Furthermore, *OsBRXL4*, *OsHSFA2d* and *OsLPA1* act as the upstream of *OsLA1* to regulate rice tiller angle (Wang et al. 2022). *OsBRXL4* can control rice tiller angle by preventing nuclear localization of OsLA1 (Li et al. 2019). HEAT STRESS TRANSCRIPTION FACTOR 2D (HSFA2D) can regulate the tiller angle by positively regulating *OsLA1* (Zhang et al. 2018). *OsLPA1* serves as the upstream of *OsBRXL4* and *OsHSFA2d* to decrease tiller angle by regulating the sedimentation rate of amyloplasts (Wu et al. 2013; Wang et al. 2022).

In this study, we identified and characterized a new member *OsNAC103* of *NAC* family, and showed that it plays critical roles in leaf senescence and plant architecture in rice. We analyzed the expression patterns of *OsNAC103* and the transcriptional activity and subcellular localization of OsNAC103 in detail. Further transgenic plants revealed that *OsNAC103*-OE lines promote leaf senescence and increase tiller angles while *osnac103* mutants delay leaf senescence and decrease tiller angles, indicating that OsNAC103 positively regulates leaf senescence and tiller angles in rice.

## Results

### OsNAC103 is a Transcription Factor of NAC Family

*OsNAC103*, annotated by Fang et al., encodes a putative transcription factor of NAC family and belongs to the SNAC (Stress-NAC) subfamily (Fang et al. 2008; Nuruzzaman et al. 2010). Phylogenetic tree analysis showed that SNAC proteins in rice and *Arabidopsis* were divided into two groups: SNAC-A and SNAC-B (Additional file 1: Fig. S1A). OsNAC103 belongs to the SNAC-B group and is highly homologous to OsNAP, ONAC016, OsNAC10, ONAC131, AtNAP and ANAC047 (Additional file 1: Fig. S1A). OsNAP and AtNAP have been identified to involve in the regulation of leaf senescence in rice and *Arabidopsis* respectively (Zhou et al. 2013; Yang et al. 2014; Liang et al. 2014). ONAC131 and OsNAC10 were revealed to participate in blast response and drought stress (Jeong et al. 2010; Sun et al. 2013). Sequence alignment in the SNAC-B group showed that there are five highly conserved regions from (a) to (e), which are the candidate regions of the NAC domain (Additional file 1: Fig. S1B). According to known members of NAC family, the five conserved regions of NAC domain play a critical role in the function of NAC proteins.

**Fig. 1:**
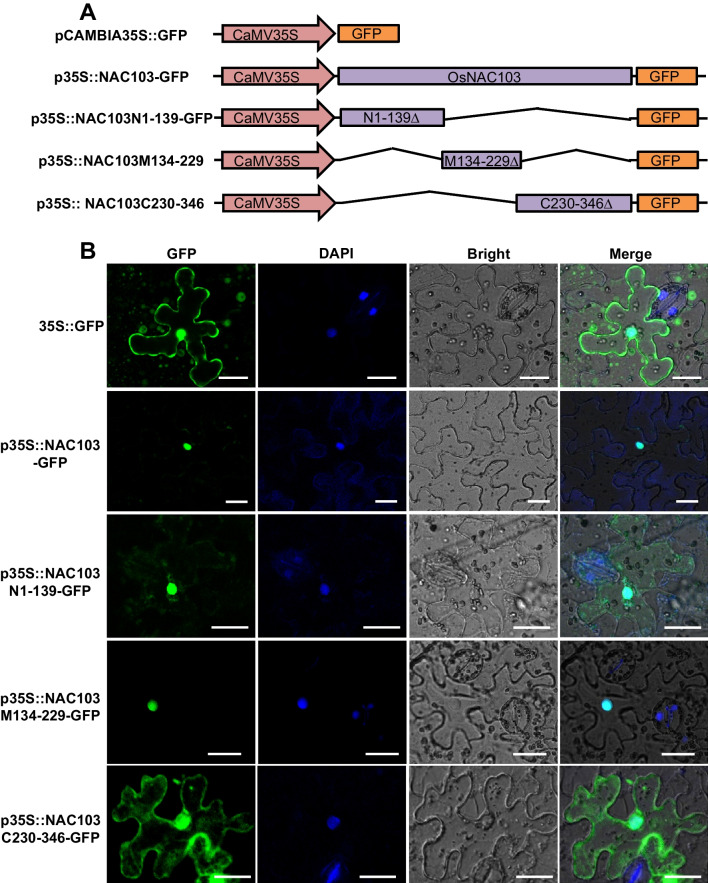
Subcellular localization of OsNAC103. **A** Diagram of the fused expression vectors. CaMV35S means the 35S promoter of Cauliflower mosaic virus. N1-139∆, M134-229∆ and C230-346∆ mean the different truncations of OsNAC103. Broken lines mean deleted parts compared with the full-length of OsNAC103. **B** Subcellular localization of OsNAC103 and its truncations in tobacco. First row: the transient expression of pCAMBIA35S::GFP empty vector. Second row: the transient expression of the full-length OsNAC103 and GFP fusion vector. Third to fifth rows: the transient expression of different truncations of OsNAC103 and GFP fusion vector. Bars = 25 μm

To confirm whether OsNAC103 has an activity of transcription factor, we firstly investigated the subcellular localization of OsNAC103, the pCAMBIA35S::OsNAC103-GFP fusion vector (Fig. 1A) and empty vector were introduced into tobacco (*Nicotiana tabacum*) leaves for transient expression. The result indicated that the fluorescent signals of OsNAC103-GFP fusion overlapped with the DAPI staining signal of the nucleus (Fig. 1B), suggesting that OsNAC103 was localized in the cell nucleus. We further constructed fused vectors with the different truncations of OsNAC103 and GFP for transient expression to reveal the nuclear localization regions of OsNAC103 (Fig. 1A). The result showed that the fusion constructs of OsNAC103N1-139-GFP (the fusion of N terminal 1–139 aa region of OsNAC103 and GFP) and OsNAC103M134-229-GFP (the fusion of 134–229 aa region of OsNAC103 and GFP) displayed significant fluorescent signals in the nucleus, while the fusion construct of OsNAC103C230-346-GFP (the fusion of C terminal 230–346 aa region of OsNAC103 and GFP) displayed fluorescent signals in the cytoplasm and nucleus, similar to the signal of empty vector (Fig. 1B), indicating that OsNAC103 contains more than one nuclear localization signals (NLS) in the N terminal 1–229 aa region.

**Fig. 2:**
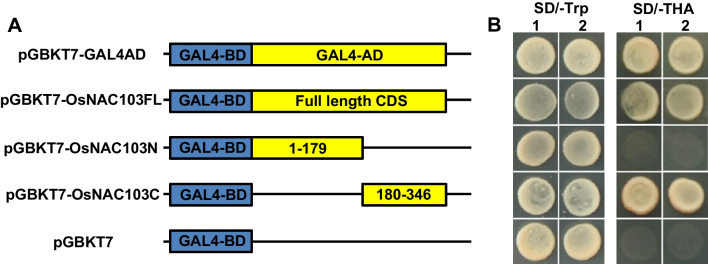
Transcriptional activation assay of OsNAC103. **A** Illustration of fusion vectors with different truncations of OsNAC103 and GAL4-BD. GAL4-BD and GAL4-AD represent the DNA-binding and activation domain of GAL4 in *Saccharomyces cerevisiae*, respectively. CDS represents the full-length CDS sequence of OsNAC103, 1–179 and 180–346 represent the truncated fragments of OsNAC103. **B** The assay of transcriptional activation of OsNAC103. Yeasts with different fusion vectors grow in single and triple deficient medium. 1 and 2 are two independent repeats. SD/-Trp represents the single-deficiency medium lacking tryptophan (Trp). SD/-THA represents the triple-deficiency medium lacking tryptophan (T), histidine (H), and adenine (A)

To further reveal whether OsNAC103 possesses the transcriptional activity, the fusion constructs pGBKT7-OsNAC103FL (the full length CDS of OsNAC103), pGBKT7-OsNAC103N (the truncation of N terminal of OsNAC103), pGBKT7-OsNAC103C (the truncation of C terminal of OsNAC103) and pGBKT7-GAL4AD (the positive control contained GAL4 AD domain) (Fig. 2A) were transformed into yeast strain Y2HGold for expression. The result showed that the transformants carrying pGBKT7-GAL4AD, pGBKT7-OsNAC103FL and pGBKT7-OsNAC103C were able to grow normally whereas the transformants carrying pGBKT7 empty (the negative control) and pGBKT7-OsNAC103N couldn’t grow on SD/-Trp/-His/-Ade medium (Fig. 2B), indicating that OsNAC103 has a transcriptional activity and possesses transcription regulatory domain (TRD) localized to the C-terminal of OsNAC103. Taken together, OsNAC103 is a transcription factor with NLS in the N-terminal and TRD in the C-terminal.

### *OsNAC103* mRNA Level is Induced by Leaf Senescence

**Fig. 3:**
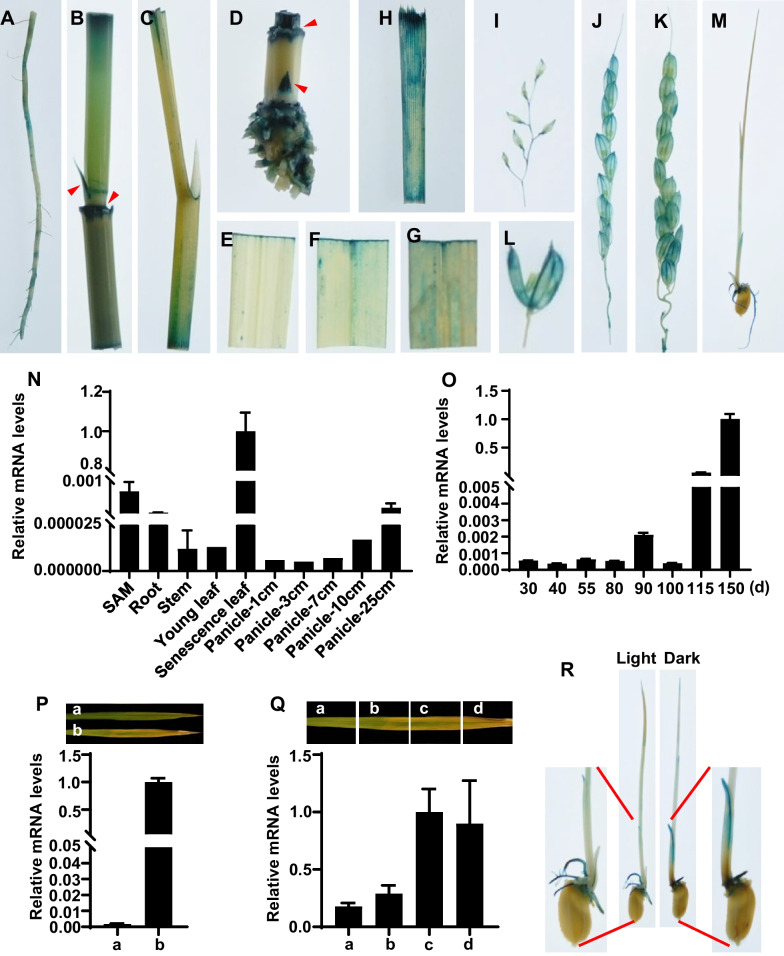
Expression patterns of *OsNAC103*. **A**–**M** GUS staining of different tissues and organs in the transgenic plants with the fused construct of *OsNAC103* promoter and *GUS* reporter. **A** Root. **B** Stem with tiller bud and intercalary meristem (indicated by arrowhead). **C** Leaf and leaf sheathe with paraphyll. **D** Root base with tiller bud and intercalary meristem (indicated by arrowhead). **E** Young leaf. **F** Mature leaf. **G** Senescent leaf. **H** Leaf sheath. **I**–**K** 5 cm, 15 cm and 20 cm young panicles, respectively. **L** Floral organ. **M** Seedling. **N** *OsNAC103* expression levels in different tissues by qRT-PCR. SAM is shoot apical meristem. **O** *OsNAC103* expression levels in leaves of different developmental stages in the field. **P** *OsNAC103* expression levels in different leaf ages. a and b represent the second and fifth leaf of the rice plant from top down, respectively. **Q** *OsNAC103* expression levels in truncated parts at the same leaf. a-d represent the four truncated parts of the fifth leaf, respectively. **R** GUS staining of seedlings at dark treatment

To investigate the spatial expression pattern of *OsNAC103*, we constructed a fusion plasmid with a 2189-bp promoter region of *OsNAC103* to drive *GUS* reporter gene, and obtained the transgenic plants to analyze the expression of *GUS* reporter gene. GUS staining assay showed that the promoter of *OsNAC103* could be activated in different tissues such as root, stem, tiller bud, leaf, leaf sheath, the different stages of panicle, floral organ and seedling (Fig. 3A–M), with the stronger expression in tiller bud and intercalary meristem (Fig. 3B, D indicated by arrowheads). For different stages of leaf, *OsNAC103* displayed higher expression in senescent leaves than that in young leaves (Fig. 3E–G). For different stages of panicle, the expression of *OsNAC103* mainly concentrated in the palea and lemma with increased levels successively in 5, 15 and 20 cm panicles (Fg. 3I–K), but almost no expression in the pistil and stamen (Fig. 3L). Further qRT-PCR analysis was in agreement with GUS staining assay, which exhibited highly expressed in senescent leaf, mature panicle (25 cm panicle) and shoot apical meristem (SAM) (Fig. 3N). We also examined the expression of *OsNAC103* at different developmental stages in leaves after sowing 30, 40, 55, 80, 90, 100, 115 and 150 days using qRT-PCR, and found that the mRNA levels of *OsNAC103* remained low from vegetative stage to reproductive stage, then significantly elevated in ripening stage (Fig. 3O).

Based on the above results, we speculated that the expression of *OsNAC103* can be induced by leaf senescence. To confirm this, we firstly analyzed the expression of *OsNAC103* in different leaf ages (the second and fifth leaf of the rice plant from top down), and found that *OsNAC103* displayed higher expression levels in older leaf (the fifth leaf) compared with the younger leaf (the second leaf) (Fig. 3P). Secondly, we detected *OsNAC103* mRNA levels of different truncated parts (indicated a-d in Fig. 3Q) at the same leaf, and the result showed that *OsNAC103* displayed a higher expression in the yellowing sectors (c and d in Fig. 3Q) compared with the green sectors (a and b in Fig. 3Q). Finally, dark treatment can induce leaf senescence, we therefore analyzed the expression of *OsNAC103* in transgenic seedlings with *proOsNAC103*::*GUS* construct after dark treatment through GUS staining. The result showed that seedlings grown under dark condition were stained more deeply in the root and coleoptile than those grown under normal light condition (Fig. 3R), suggesting that *OsNAC103* has a higher expression in the dark condition. Taken together, *OsNAC103* mRNA level is induced by leaf senescence and OsNAC103 may play an important role in leaf senescence of rice.

### *OsNAC103* expression is induced by different phytohormones and abiotic stresses

**Fig. 4:**
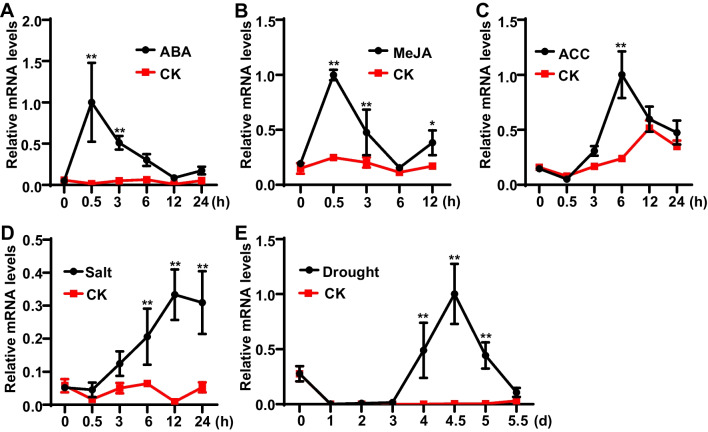
*OsNAC103* is induced by ABA, MeJA, ACC and abiotic stresses. **A**–**C** *OsNAC103* expressions under the ABA, MeJA and ACC treatments, respectively. 30 days seedlings were treated with the 100 μM ABA, MeJA and ACC by spraying, and RNAs were isolated at indicated times to perform expression analysis using qRT-PCR. **D**, **E** *OsNAC103* expression under high salinity and drought treatments, respectively. 30 days seedlings were treated with the 200 mM NaCl and drought, and RNAs were isolated at indicated times to perform expression analysis using qRT-PCR. CK means the seedlings without the treatment of ABA, MeJA, ACC, NaCl and drought under normal condition. Asterisks indicate statistically significant differences by Student’s t test (*P < 0.05; **P < 0.01)

Given that leaf senescence can be induced by different phytohormones and abiotic stresses such as ABA (Abscisic acid), MeJA (Methyl jasmonate), ACC (1-aminocyclopropane-1-carboxylic acid, ethylene precursor) phytohormones and high salinity, drought stresses, we analyzed *OsNAC103* expression levels under the treatments of phytohormones and abiotic stresses using qRT-PCR. The results showed that the expression of *OsNAC103* was significantly up-regulated under these treatments (Fig. 4A–E). For ABA and MeJA treatments, the expression levels of *OsNAC103* were induced rapidly and reached peaks at the 0.5 h after treatments (Fig. 4A, B). For ACC, the expression of *OsNAC103* was induced after 3 h and displayed a peak at the 6 h (Fig. 4C). For high salinity stress, *OsNAC103* expression was gradually up-regulated and reached a peak at the 12 h (Fig. 4D). For drought stress, *OsNAC103* expression was induced after 4 days and displayed a peak at 4.5 days (Fig. 4E). Collectively, above results showed that *OsNAC103* expression was induced by ABA, MeJA, ACC as well as high salinity and drought stresses.

### OsNAC103 Positively Regulates Leaf Senescence in Rice

To explore the function of OsNAC103, we generated the transgenic plants overexpressing *OsNAC103* and CRISPR-Cas9 editing mutants. For overexpression plants, we selected five independent lines (OE1, OE2, OE6, OE7 and OE9) to detect mRNA levels of *OsNAC103* using qRT-PCR, and found that *OsNAC103* mRNA levels were significantly elevated in those lines (Additional file 1: Fig. S2B). For CRISPR-Cas9 editing mutants, we designed three CRISPR-Cas9 targets located at the first exon, second exon and third exon of *OsNAC103* (Additional file 1: Fig. S3A), and fused with sgRNA respectively. Then three fragments with target and sgRNA were simultaneously inserted into pCAMBIA1300 binary vector with Cas9 to obtain the recombinant vector which were used to generate transgenic plants. We finally obtained two homozygous *osnac103* mutants (CR2 and CR5) by sequencing analysis. CR2 contained 1-bp bi-allelic insertion in target 1, 3-bp deletion in target 2 and 1-bp bi-allelic insertion in target 3, respectively (Additional file 1: Fig. S3B–D). CR5 contained 1-bp insertion in target 1, 1-bp insertion in target 2 and 1-bp deletion in target 3, respectively (Additional file 1: Fig. S3E–G). Both CR2 and CR5 resulted in the frameshift of amino acids due to insertions and/or deletions.

**Fig. 5:**
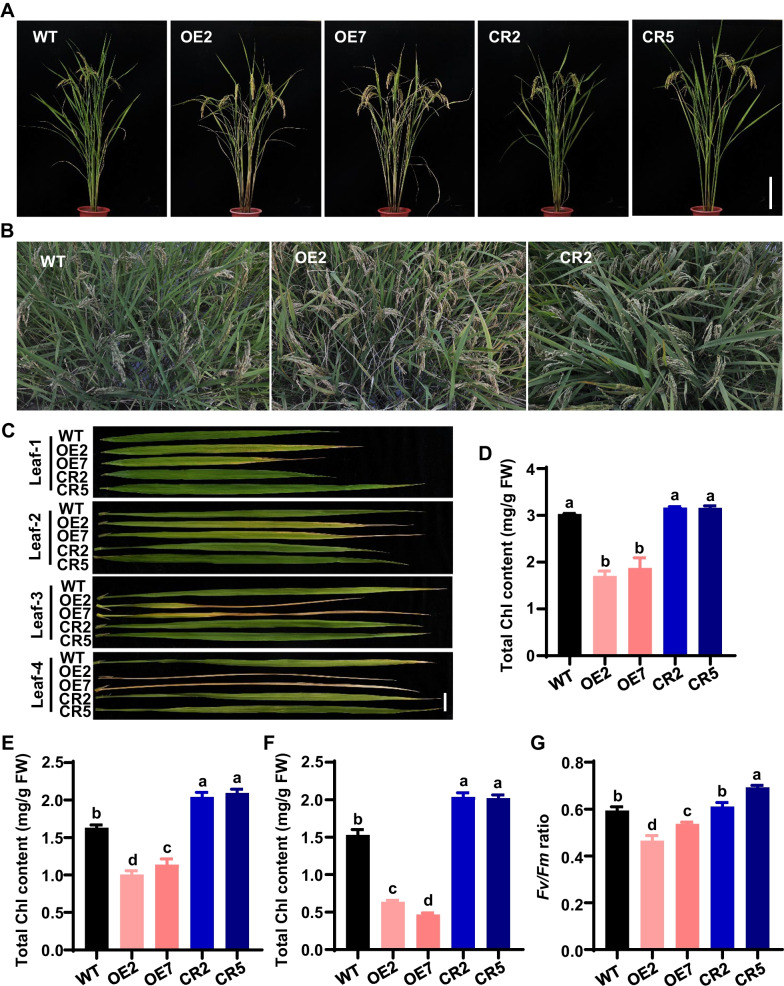
OsNAC103 positively regulates leaf senescence in rice. **A**–**C** Phenotype of *OsNAC103*-OE lines and *osnac103* mutants during the reproductive growth stage. **A** Phenotype of *OsNAC103*-OE plants and *osnac103* plants. Bar = 20 cm. **B** Phenotype of *OsNAC103*-OE lines and *osnac103* mutants in the field. **C** Phenotype of different leaves in WT, *OsNAC103*-OE lines and *osnac103* mutants. Leaf-1, Leaf-2, Leaf-3, Leaf-4 represent the first, second, third and fourth leaves of the rice plant from top down, respectively. Bar = 2 cm. **D**–**F** Total chlorophyll contents of the flag leaves, penultimate leaves and antepenultimate leaves in *OsNAC103*-OE lines and *osnac103* mutants, respectively. **G** Fv/Fm ratio of *OsNAC103*-OE lines and *osnac103* mutants in penultimate leaves. OE2 and OE7 are two independent *OsNAC103*-OE lines. CR2 and CR5 are two allelic mutants. The letters a, b, c, d indicate significant differences from the one-way ANOVA analysis (*P* < 0.05)

Phenotypic observation found that OE1, OE6 and OE9 with higher expression levels of *OsNAC103* displayed extremely pleiotropic phenotypes with premature senescent and droopy leaves, large tiller angles and reduced plant height in T_0_ generation (Additional file 1: Fig. S2A), and we failed to gain the seeds due to lethal effect during vegetative growth stage in OE1, OE6 and OE9. OE2 and OE7 lines with relative lower expression levels of *OsNAC103* displayed the lighter phenotypes with leaf senescence, increased tiller angles and decreased plant height in T_0_ generation (Additional file 1: Fig. S2A), and these lines could normally grow and harvest seeds. We therefore used OE2 and OE7 lines as well as CR2 and CR5 mutants to further analyze their phenotypes in T_2_ generation. The result showed that OE2 and OE7 displayed markedly phenotype of leaf senescence during the reproductive growth stage (Fig. 5A–C), while two mutants and WT presented normal phenotype in leaves (Fig. 5A–C), even much greener in CR2 and CR5 mutants than in WT (Fig. 5A–C). Total Chl contents in different leaves from flag leaf to antepenultimate leaf were measured, and the result demonstrated that Chl contents of OE2 and OE7 were significantly lower than those of WT and two mutants in flag leaves, penultimate leaves and antepenultimate leaves (Fig. 5D–F), whereas Chl contents of CR2 and CR5 were significantly higher than those of WT in penultimate leaves and antepenultimate leaves (Fig. 5E, F), suggesting that overexpression of *OsNAC103* promotes leaf senescence while mutation of *OsNAC103* delays leaf senescence. Fv/Fm ratio (efficiency of PSII) can reflect the efficiency of photosynthesis, we therefore measured the Fv/Fm ratio after heading in the field. The result showed that Fv/Fm ratio significantly decreased in OE2 and OE7 lines while increased in CR2 and CR5 mutants (Fig. 5G), indicating that *OsNAC103* can affect the efficiency of photosynthesis during leaf senescence.

During the vegetative growth stage, the top leaves including flag leaves, penultimate leaves and antepenultimate leaves were no significant difference in WT, two OE lines and two mutants (Additional file 1: Fig. S4A–C), however, the bottom leaves (e.g. the fourth and fifth leaves) exhibited premature-senescence phenotype in OE2 and OE7 lines, and Chl levels also clearly reduced compared with WT (Additional file 1: Fig. S4A–E). Importantly, the bottom leaves in CR2 and CR5 mutants were much greener compared with WT and OE lines, and Chl levels were also higher than those of WT and OE lines (Additional file 1: Fig. S4A–E), indicating that *OsNAC103* might accelerate the degradation of Chl and promote leaf senescence.

### OsNAC103 Can Regulate Plant Architecture in Rice

**Fig. 6:**
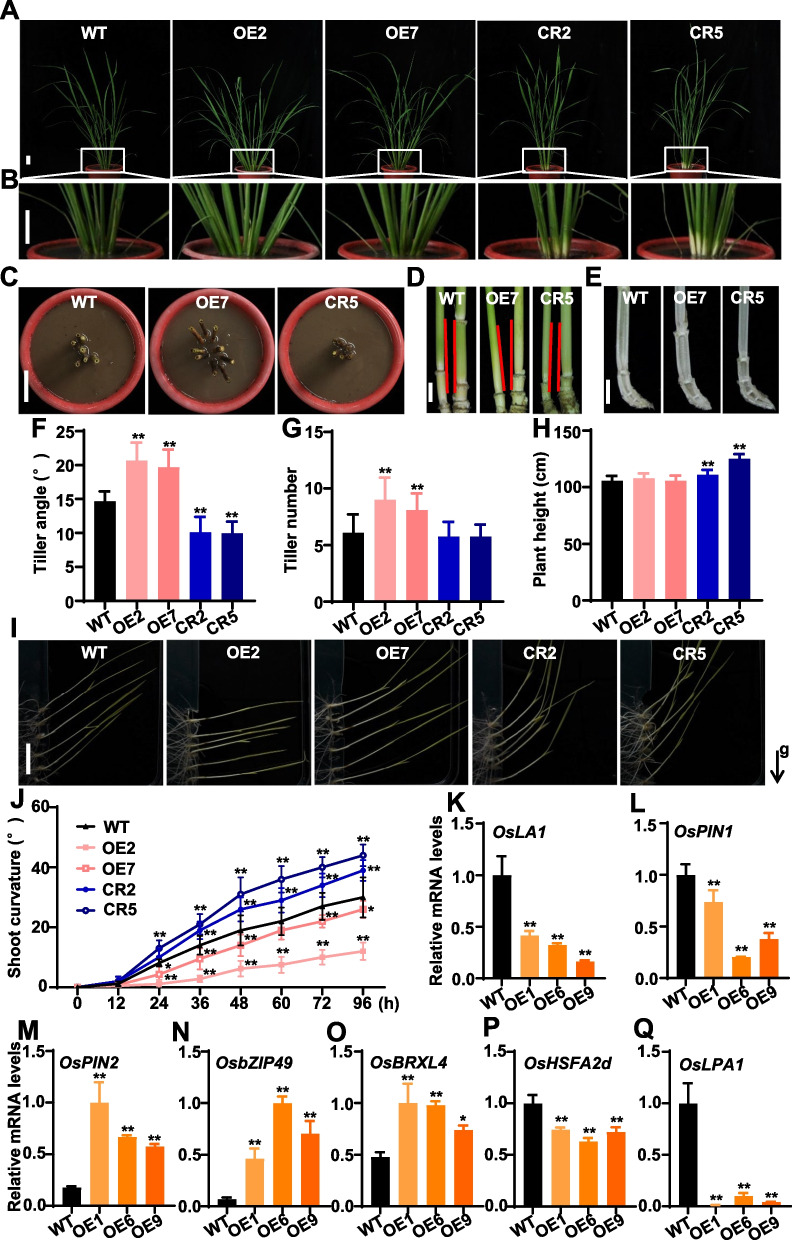
*OsNAC103* regulates the plant architecture in rice. **A**, **B** Phenotype of tiller angles of *OsNAC103*-OE lines and *osnac103* mutants in vegetative stage. B indicates the magnified figure in A. Bars = 5 cm. **C**–**E** Phenotype of the tiller base (TB) of *OsNAC103*-OE lines and *osnac103* mutants in reproductive stage. Bar = 5 cm in **C**, Bars = 1 cm in **D** and **E**. **F** Tiller angles of *OsNAC103*-OE lines and *osnac103* mutants. **G** Tiller number of *OsNAC103*-OE lines and *osnac103* mutants. **H** Plant height of *OsNAC103*-OE lines and *osnac103* mutants. Values are shown as means ± SD, n ≥ 20 individual plants. **I** Shoot gravitropism of *OsNAC103*-OE lines and *osnac103* mutants grown under dark condition. Photos were taken at 96 h after rotation 90 degrees. Bar = 2 cm. g means the direction of gravity. **J** Dynamics of shoot gravitropism grown under dark condition. The shoot curvatures are measured in 12 h interval (n = 9–13). **K**–**Q** Expression analysis of genes associated with tiller angle and shoot gravitropism in *OsNAC103*-OE lines. RNAs were extracted from penultimate leaves of WT and OE lines at vegetative stage, and qRT-PCR was performed to analyze the relative expression levels of different genes. OE1, OE6 and OE9 are three independent lines with higher overexpression levels and extremely pleiotropic phenotypes. OE2 and OE7 are two independent *OsNAC103*-OE lines. CR2 and CR5 are two allelic mutants. Asterisks indicate statistically significant differences by Student’s t test (*P < 0.05; **P < 0.01)

In addition to the phenotype of leaf senescence, OE2 and OE7 displayed loose plant architecture with larger tiller angles during the vegetative and reproductive growth stages (Fig. 6A–C). Observation for the tiller base in detail showed that the inclined angles between the main culm and its first side tiller in OE lines were larger than those in WT and* osnac103* mutants (Fig. 6D), and more symmetrical growth of the tiller node was observed in OE lines compared with WT and *osnac103* mutants (Fig. 6E). We further measured the tiller angle at heading stage and found that the tiller angles in OE2 and OE7 (the mean values are 20.65 and 19.65, respectively) were larger than those of WT (the mean value is 14.65), while the tiller angles in CR2 and CR5 (the mean values are 10.1 and 9.95, respectively) were smaller than those of WT (Fig. 6F). Moreover, the tiller numbers of OE lines were significantly more than those of WT and *osnac103* mutants (Fig. 6G). The plant height of CR2 and CR5 were significantly higher than those of WT and OE lines (Fig. 6H). The panicle length and the number of primary branches in OE lines and *osnac103* mutants were no significant differences compared with WT (Additional file 1: Fig. S5A, B), while the weight of 100 grains in OE lines were significantly decreased compared with WT (Additional file 1: Fig. S5C), suggesting that OsNAC103 may influence yield due to leaf senescence. Given that tiller angle is closely associated with negative gravitropism of shoot, we examined the gravitropic response in WT, OE lines and *osnac103* mutants. Compared with WT, the shoot curvatures of OE2 and OE7 lines were reduced, while were increased in CR2 and CR5 mutants after the treatment of shoot gravitropism (Fg. 6I, J), indicating that the gravitropic response was reduced in OE lines but increased in *osnac103* mutants. These results demonstrated that *OsNAC103* can regulate rice tiller angle in plant architecture through negative gravitropism of shoot.

To further elucidate the regulatory function of *OsNAC103* in rice tiller angle, we examined the expression levels of genes associated with tiller angle and shoot gravitropism in OE lines (OE2 and OE7) and *osnac103* mutants, and found that only *OsLA1* expression displayed significant difference in OE2 and OE7 compared with WT (Additional file 1: Fig. S6A). The remaining genes including *OsBRXL4*, *OsHSFA2d* and *OsLPA1* had no significant difference in OE lines and *osnac103* mutants compared with WT (Additional file 1: Fig. S6B–D). Given that different OE lines exhibited different overexpression levels (Additional file 1: Fig. S2B), we investigated the expression levels of these genes in OE1, OE6 and OE9 with higher overexpression levels and extremely pleiotropic phenotypes. The result showed that *OsLA1* was significantly down-regulated in OE1, OE6 and OE9 compared with WT (Fig. 6K), indicating that *OsNAC103* regulates the tiller angle by *OsLA1*-dependent pathway. We further analyzed the expression of *OsPIN1* and *OsPIN2* which act as the downstream of *OsLA1* to regulate auxin transport asymmetric auxin distribution (*OsPIN1* and *OsPIN2* displayed the contrary effect on the auxin transport) in three OE lines, and found that *OsPIN1* was significantly down-regulated and *OsPIN2* was significantly up-regulated in OE1, OE6 and OE9 (Fig. 6L, M), indicating that *OsNAC103* may serve as the upstream of *OsLA1*, *OsPIN1* and *OsPIN2* to regulate tiller angle. Also, *OsbZIP49*, an important transcription factor modulating tiller angle by regulating local auxin homeostasis, was significantly up-regulated in OE1, OE6 and OE9 (Fig. 6N), indicating that OsNAC103 can indeed affect auxin distribution to regulate tiller angle in rice. *OsBRXL4*, *OsHSFA2d* and *OsLPA1* act as the upstream of *OsLA1* to regulate rice tiller angle (see the introduction). Thus, we also detected the expression of *OsBRXL4*, *OsHSFA2d* and *OsLPA1* in three OE lines. The result showed that *OsBRXL4* was significantly up-regulated and *OsHSFA2d* and *OsLPA1* were significantly down-regulated in OE1, OE6 and OE9 (Fig. 6O–Q), implying that OsNAC103 can regulate the expression levels of the upstream of *OsLA1* to modulate rice tiller angle. Collectively, OsNAC103 can regulate the tiller angle by *OsLA1*-dependent pathway.

### *osnac103* Mutants Display Stay-Green Phenotype Under Dark-Induced Senescence

**Fig. 7:**
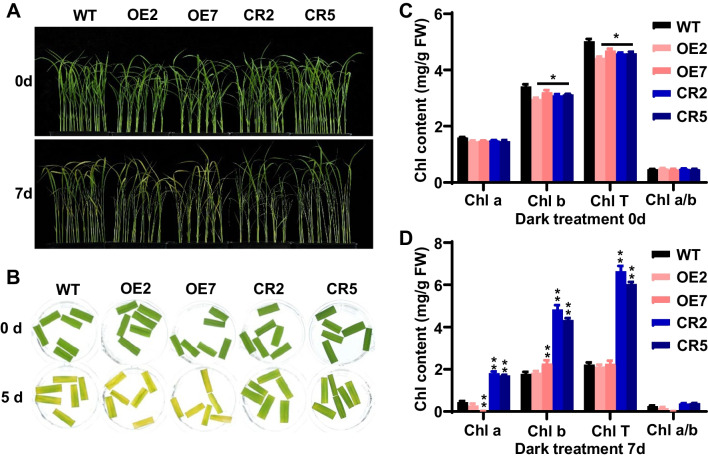
*osnac103* mutants display green-stay phenotype under dark-induced senescence. **A** Phenotype of *OsNAC103*-OE lines and *osnac103* mutants under the dark treatment. **B** Phenotype of detached leaves of *OsNAC103*-OE lines and *osnac103* mutants under the dark treatment. **C**, **D** Chlorophyll content analysis of leaves in *OsNAC103*-OE lines and *osnac103* mutants before dark treatment (**C**) and after dark treatment (**D**). The leaves with same weight before dark treatment and after dark treatment 7 days were collected to measure Chl content. OE2 and OE7 are two independent *OsNAC103*-OE lines. CR2 and CR5 are two allelic mutants. Values are shown as means ± SD, n = 3. Asterisks indicate statistically significant differences by Student’s t test (*P < 0.05; **P < 0.01)

To study the regulation of leaf senescence by OsNAC103 in more detail, we examined the phenotype of *OsNAC103*-OE lines and *osnac103* mutants under dark-induced senescence (DIS). Three-week-old plants were transferred to darkness, and the phenotype was observed and recorded after 7 days of dark incubation (DDI). The result showed that OE2, OE7 and WT leaves became yellowish, while CR2 and CR5 leaves retained much greener, displaying stay-green phenotype (Fig. 7A). The stay-green phenotype of CR2 and CR5 was further confirmed using detached leaf discs (Fig. 7B), we could clearly see that detached leaf discs of CR2 and CR5 were greener than those of WT and OE lines after 5 DDI (Fig. 7B), indicating that mutation of *OsNAC103* attenuates dark-induced leaf senescence. The measurement of Chl levels showed that Chl a, Chl b, and the total Chl levels were largely consistent in WT, OE lines and *osnac103* mutants, with slightly higher Chl levels in WT before dark treatment (Fig. 7C). However, Chl a, Chl b and total Chl levels in CR2 and CR5 mutants were significantly higher than those in WT and OE lines after dark treatment (Fig. 7D), suggesting that mutation of *OsNAC103* inhibits Chl degradation. Taken together, *OsNAC103* can positively regulate leaf senescence under DIS condition, in agreement with in the natural senescence.

### *OsNAC103* Promotes ABA and JA-Induced Leaf Senescence

**Fig. 8:**
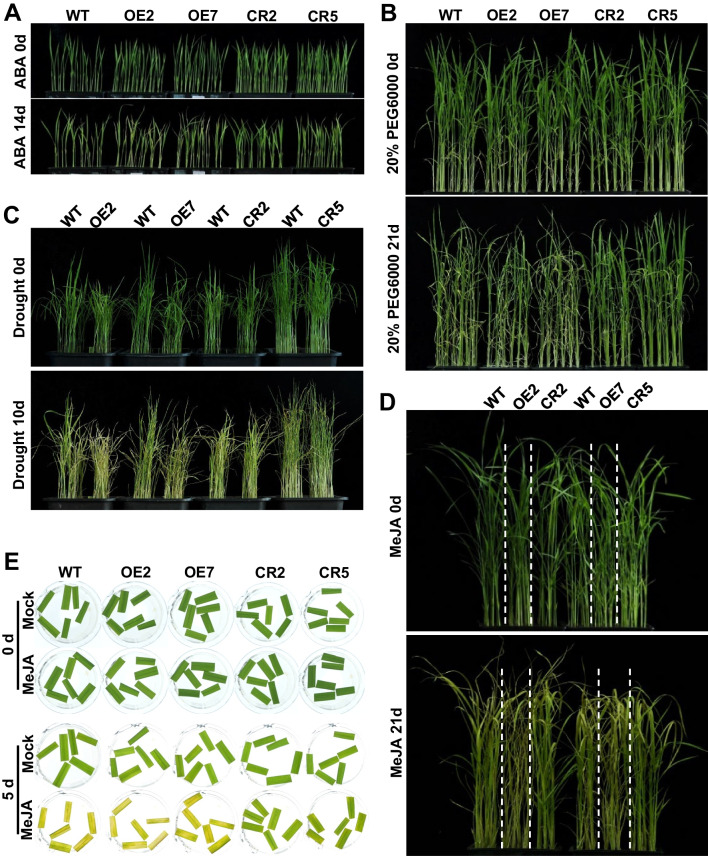
*OsNAC103* promotes ABA and JA-induced leaf senescence. **A**–**D** Phenotypes of *OsNAC103*-OE lines and *osnac103* mutants under the treatments of 100 μM ABA (**A**), 20% PEG6000 (**B**) drought (**C**) and 100 μM MeJA (**D**). Seedlings grown in hydroponic solution were treated with 100 μM ABA and MeJA or with 20% PEG6000. For drought treatment, seedlings grown in soil were stopped water supplement. **E** Phenotype of detached leaves of *OsNAC103*-OE lines and *osnac103* mutants under 100 μM MeJA treatment. OE2 and OE7 are two independent *OsNAC103*-OE lines. CR2 and CR5 are two allelic mutants

Given that *OsNAC103* mRNA level was induced by ABA and MeJA, we analyzed the response of OE lines and *osnac103* mutants under the treatments of ABA and MeJA. Seedlings grown in hydroponic nutrient solution were treated with 100 μM ABA and MeJA, respectively. After two weeks, for ABA treatment, we found that the yellowish phenotype of CR2 and CR5 was significantly slower than that of WT, while displayed contrary effect on OE2 and OE7 lines (Fig. 8A). ABA is usually associated with drought stress to promote leaf senescence, we therefore performed simulated drought stress using PEG6000 treatment. After three weeks of treatment, we found that CR2 and CR5 exhibited much greener phenotype than WT and OE lines (Fig. 8B). We also performed the experiment of drought treatment using the seedling grown in soil by stopping water supplement in WT, OE lines and CR mutants. After ten days of treatment, we found that CR2 and CR5 exhibited much greener phenotype than WT and OE lines (Fig. 8C), similar to the phenotype of PEG6000 and ABA treatments. For MeJA treatment, the yellowish phenotype of OE2 and OE7 was significantly faster than WT, but displayed contrary effect on CR2 and CR5 (Fig. 8D). We also used detached leaf discs to confirm the phenotype under MeJA treatment, and the result was consistent with the result of hydroponic condition (Fig. 8E). Taken together, these results suggested that *OsNAC103* accelerates ABA and JA-induced leaf senescence.

### *OsNAC103* Regulates the Expression of Genes Associated with Senescence, ABA and JA Pathway

**Fig. 9:**
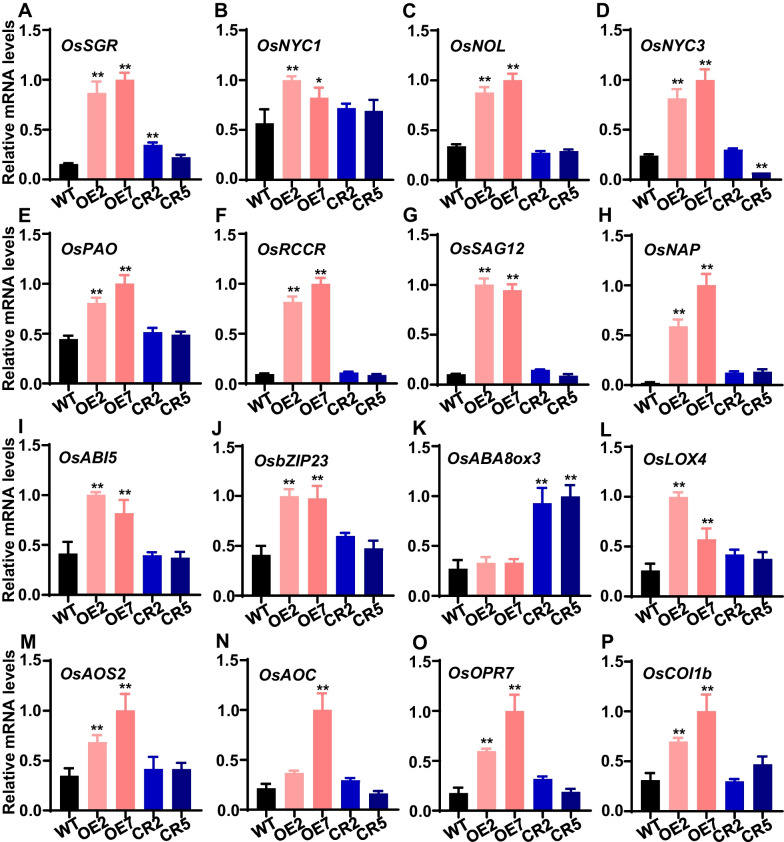
Expression of senescence-associated genes and ABA, JA pathway-associated genes in *OsNAC103*-OE lines and *osnac103* mutants. **A-H** Expression of senescence-associated genes in *OsNAC103*-OE lines and *osnac103* mutants. **I-K **Expression of ABA pathway-associated genes in *OsNAC103*-OE lines and *osnac103 *mutants. **L-P** Expression of JA pathway-associated genes in *OsNAC103*-OE lines and* osnac103* mutants. RNAs were extracted from penultimate leaves of WT, OE lines and *osnac103* mutants at vegetative stage, and qRT-PCR was performed to analyze the relative expression levels of different genes. OE2 and OE7 are two independent *OsNAC103*-OE lines. CR2 and CR5 are two allelic mutants. Asterisks indicate statistically significant differences by Student’s t test (*P < 0.05; **P < 0.01)

To further elucidate the regulatory function of *OsNAC103* in rice leaf senescence, we examined the expression levels of genes associated with leaf senescence pathway in *OsNAC103*-OE lines and *osnac103* mutants. The result showed that these key senescence identity genes including *OsSGR*, *OsNYC1*, *OsNOL*, *OsNYC3*, *OsPAO*, *OsRCCR*, *OsSAG12* and *OsNAP* were significantly up-regulated in OE2 and OE7 lines (Fig. 9A–H), indicating that *OsNAC103* positively regulates the expression of *SAGs* to modulate leaf senescence in rice. Given that *OsNAC103* is involved in ABA and JA-induced leaf senescence, we also investigated the expression levels of key genes associated with ABA and JA pathways in *OsNAC103*-OE lines and *osnac103* mutants. The result showed that *OsABI5* and *OsbZIP23* associated with ABA signal pathway were significantly up-regulated in OE2 and OE7 compared with WT (Fig. 9I, J). *OsABA8ox3* associated with ABA degradation pathway was significantly up-regulated in CR2 and CR5 compared with WT (Fig. 9K). Meanwhile, *OsLOX4*, *OsAOS2*, *OsAOC* and *OsOPR7* associated with JA synthesis pathway were significantly up-regulated in OE2 and OE7 compared with WT (Fig. 9L–O). *OsCOI1b* associated with JA signal pathway was significantly up-regulated in OE2 and OE7 compared with WT (Fig. 9P). These results indicate that *OsNAC103* can regulate the expression of ABA and JA pathway-associated genes to modulate leaf senescence in rice.

## Discussion

### *OsNAC103* Can Positively Regulate Leaf Senescence in Rice

Leaf senescence is regulated by both internal gene networks and external environmental factors. In the regulatory gene networks of leaf senescence in plants, lager number of TFs are involved in these processes and play crucial roles. The plant-specific NAC TFs have been reported that played positive or negative roles in the regulation of leaf senescence. For examples, ONAC106 and OsNAC109 are two senescence-inhibiting TFs (Sakuraba et al. 2015; Li et al. 2021). AtNAP in *Arabidopsis* (Guo and Gan 2006; Kou et al. 2012; Zhang and Gan 2012; Yang et al. 2014), and OsNAP, OsNAC2, ONAC096 and ONAC054 in rice were acted as senescence-promoting TFs (Zhou et al. 2013; Liang et al. 2014; Mao et al. 2017; Kang et al. 2019; Sakuraba et al. 2020). In this study, we identified a novel NAC family member, OsNAC103, playing an important role in the regulation of rice leaf senescence. Our results showed that OsNAC103 had a transcriptional activity and was localized in the nucleus (Figs. 1 and 2), suggesting that OsNAC103 serves as a transcription factor. Expression analysis indicated that *OsNAC103* mRNA level was dramatically induced in senescent leaves (Fig. 3E–G, N–Q), demonstrating that *OsNAC103* is involved in the regulation of leaf senescence. Further genetic evidences showed that overexpression of *OsNAC103* promoted leaf senescence and the mutation of *OsNAC103* delayed leaf senescence under natural condition and DIS condition (Figs. 5A–C and 7A, B). Meanwhile, the expression of *SAGs* was up-regulated in *OsNAC103-*OE lines (Fig. 9A–H). These results confirmed that OsNAC103 can positively regulate leaf senescence in rice.

In addition, *OsNAC103* mRNA levels were induced by different phytohormones and abiotic stresses such as ABA, MeJA and drought (Fig. 4A, B, E). Further experiments confirmed that OsNAC103 promotes ABA, JA and stress-induced leaf senescence (Figs. 7 and 8), indicating that OsNAC103 may regulate leaf senescence through ABA and JA signal pathways. In *Arabidopsis*, *AtNAP* was induced by ABA and could directly bind to the promoter region of *SAG113* to regulate ABA-mediated stomatal movement and water loss during leaf senescence (Zhang and Gan 2012). In rice, OsNAC2 bound directly to the promoters of ABA biosynthesis genes *OsNCED3* and *OsZEP1* and ABA catabolic gene *OsABA8ox1* to lead to an increase in ABA levels, promoting leaf senescence (Mao et al. 2017). ONAC054 could bind the promoters of *OsABI5* and *OsABF4*, key genes of ABA signal pathway, to up-regulate their expression, thus regulated ABA-induced leaf senescence (Sakuraba et al. 2020). *ONAC096* could up-regulate the expression levels of *OsABI5* and *OsEEL* to promote leaf senescence (Kang et al. 2019). In addition, OsNAP regulated leaf senescence through upregulating the expression of JA biosynthesis genes *OsLOX2*, *OsAOC* and *OsOPR7* (Zhou et al. 2013). In our result, the expression of *OsABI5*, *OsbZIP23* and *OsABA8ox3* in ABA pathway and *OsLOX4*, *OsAOS2*, *OsAOC*, *OsOPR7* and *OsCOI1b* in JA pathway were significantly up-regulated or down-regulated in *OsNAC103-*OE lines and *osnac103* mutants (Fig. 9I–P). Further yeast one hybrid analysis showed that OsNAC103 did not indirectly interact with the promoters of *SAGs* including *OsSGR*, *OsNYC1*, *OsNYC3*, *OsPAO*, *OsRCCR* and *OsSAG12* (Additional file 1: Fig. S7). Based on these results, we speculate that OsNAC103, as a transcription factor, may bind the promoters of key genes associated with ABA or JA pathways to regulate ABA or JA-induced leaf senescence. Future chromatin immunoprecipitation (ChIP) and luciferase (LUC) activity assays will provide possible evidences for their interaction.

### OsNAC103 Can Regulate the Plant Architecture in Rice

In addition to the regulatory role in leaf senescence, OsNAC103 also regulates the plant architecture in rice. *OsNAC103*-OE lines displayed loose plant architecture with larger tiller angles while tiller angles of *osnac103* mutants decreased during the vegetative and reproductive growth stages (Fig. 6A–C, F; Additional file 1: Fig. S2A). Further experiments confirmed that *OsNAC103* affected the negative gravitropism of shoot to regulate rice tiller angles (Fig. 6I, J). Meanwhile, *OsNAC103* also affected the tiller number and plant height (Fig. 6G, H). These evidences showed that OsNAC103 acts a crucial role in the plant architecture in rice. As one of the largest TF family in plants, NAC TF members serve as the pleiotropy functions in the processes of plant growth and development. ONAC106, a homolog of OsNAC103, increases the tiller angle through decreasing the expression of *OsLPA1* (Sakuraba et al. 2015). In our study, *OsLPA1* was significantly down-regulated in *OsNAC103*-OE lines. In addition, other genes associated with tiller angle and shoot gravitropism such as *OsBRXL4*, *OsbZIP49* and *OsPIN2* were significantly up-regulated while *OsHSFA2d*, *OsLA1* and *OsPIN1* were significantly down-regulated in *OsNAC103*-OE lines, indicating that *OsNAC103* controls tiller angle through the regulation of HSFA2D-LA1 pathway.

In addition, ONAC054/RIM1, another homolog of OsNAC103, also regulates the plant architecture and leaf senescence in rice (Sakuraba et al. 2020). *rim1* mutant exhibited dwarf and root growth inhibition phenotypes, suggesting that ONAC054/RIM1 can regulate the plant architecture in rice, and as a JA signaling component, ONAC054/RIM1 can regulate the expression of JA biosynthetic genes such as *OsLOX*, *OsAOS2* and *OsOPR7* to affect the plant architecture in rice (Yoshii et al. 2010). Moreover, ONAC054/RIM1 positively regulates leaf senescence, similar to the role of OsNAC103 in the regulation of leaf senescence. Taken together, OsNAC103 as well as other NAC members exhibit pleiotropy roles through regulating different downstream genes in the processes of plant growth and development.

### OsNAC103 Contains Multiple NLSs as a Transcription Factor

In this study, the transient expression assays with different truncations of OsNAC103 and GFP fusion showed that fused proteins exhibited localized signals in the nucleus, indicating that OsNAC103 protein may contain multiple NLSs in its N terminal 1–229 region (Fig. 1B). Further sequence analysis of amino acids in the N terminal 1–229 region of OsNAC103 showed that there were five highly conserved regions from (a) to (e) which are the candidate regions of NLS (Additional file 1: Fig. S1B) (Kikuchi et al. 2000; Li et al. 2021). In database of rice genome annotation project (http://rice.uga.edu/), *OsNAC103* transcript was predicted to contain three alternative splices from Loc_Os07g48450.1 to Loc_Os07g48450.3. Loc_Os07g48450.1 is full-length transcript contained three exons and two introns (Additional file 1: Fig. S8A, B). Loc_Os07g48450.2 is a truncated transcript with two exons and one intron (Additional file 1: Fig. S8A, B). Loc_Os07g48450.3 only contains one exon and one intron (Additional file 1: Fig. S8A, B). Loc_Os07g48450.2 and Loc_Os07g48450.3 transcripts can produce truncated proteins compared with Loc_Os07g48450.1 (Additional file 1: Fig. S8B). We further designed primers at the different regions of these three transcripts to confirm the possible alternative splices (Additional file 1: Fig. S8A). According to the database, Loc_Os07g48450.1, Loc_Os07g48450.2 and Loc_Os07g48450.3 should exhibit the predicted fragments of 540 bp, 620 bp and 1042 bp using primer pair F and R, respectively. Our RT-PCR result demonstrated three bands amplified from cDNA of leaves. Among three bands, 540 bp and 620 bp fragments were consistent with the prediction of Loc_Os07g48450.1 and Loc_Os07g48450.2 respectively, indicating that Loc_Os07g48450.1 and Loc_Os07g48450.2 did present (Additional file 1: Fig. S8C). We could also see that Loc_Os07g48450.1 was main splicing form with highly expression level (Additional file 1: Fig. S8C, D). Amplified ~ 400 bp band was not in agreement with the prediction of Loc_Os07g48450.3 which should obtain 1042 bp band using primer pair F and R (Additional file 1: Fig. S8C). We further designed a reverse primer indicated as R’ (located at the 326 bp downstream of R) to confirm the above result. We could see the amplified bands with primer pair F and R’ were increased ~ 300 bp (Additional file 1: Fig. S8D), in agreement with the prediction of Loc_Os07g48450.1 and Loc_Os07g48450.2. Meanwhile, we found that original ~ 400 bp band with primer pair F and R also increased and obtained ~ 700 bp band (Additional file 1: Fig. S8D), suggesting that this band should be another alternative splice but not the predicted Loc_Os07g48450.3.

Our result showed that the truncated fusion proteins with N1–139 and M134–229 clearly localized in the nucleus, but the truncated fusion protein with C230–346 was no specific signal in the nucleus (Fig. 1B). According to this result, the truncated protein produced by Loc_Os07g48450.2 should contain NLS because it comprises M134–229 region in our transient expression assays (Fig. 1; Additional file 1: Fig. S8A, B). However, Loc_Os07g48450.2 lacks most amino acids of NAC domain at N-terminal (Additional file 1: Fig. S8B). Whether Loc_Os07g48450.2 can simultaneously regulate leaf senescence and plant architecture, or only regulate leaf senescence or plant architecture, or play new roles, or no function, it still needs more evidence to confirm.

## Materials and Methods

### Plant Materials

Rice variety Zhonghua11 (*Oryza sativa* L. ssp. *japonica*), *OsNAC103* overexpression transgenic lines (*OsNAC103*-OE lines) and *osnac103* mutants by CRISPR-Cas9 system were used in this research. Transgenic plants were generated from the rice variety Zhonghua11 using the *Agrobacterium*-mediated transformation method described by Hiei et al. (1994). All plant materials were grown in the experimental field of Tianjin Normal University.

### Construction of the Vectors and Rice Transformation

For overexpressing vector, full-length cDNA fragment of *OsNAC103* (1041-bp) was amplified from 40-day-old leaves by RT-PCR using specific primers of *OsNAC103* (Additional file 2: Table S1). The resulting fragment was inserted into pCAMBIA2300 binary vector with double CaMV35S promoters to obtain the recombinant vector. For the construction of *osnac103* mutants by CRISPR-Cas9, according to the method described previously (Ma et al. 2015), *OsNAC103* targets were obtained by annealing with gene-specific primers (Additional file 2: Table S1) and inserted into sgRNA construct respectively, then the fragments with target and sgRNA were inserted into pCAMBIA1300 binary vector with Cas9 to obtain the recombinant vector. All recombinant vectors were introduced into Zhonghua11 using the *Agrobacterium*-mediated transformation method to produce corresponding transgenic plants.

### Quantitative Real-Time PCR (qRT-PCR) Analysis

Total RNA was isolated using Trizol solution (Aidlab, Beijing, China) from corresponding tissues and organs of rice plants. The cDNA was synthesized from 1 μg of total RNA using the cDNA synthesis kit with a gDNA wiper (TaKaRa, Beijing, China). One microliter of cDNA was used for qRT-PCR analysis with gene-specific primers using SYBR Green PCR master mix in a LightCycler480 system (Roche). The 2^− ΔΔCT^ method described by Livak and Schmittgen (2001) was used for the analysis of relative gene expression. Three biological replicates were performed, and *OsActin1* was used as an internal control. All primers are shown in Additional file 2: Table S1.

### *OsNAC103* Expression Profile Analysis

For GUS staining assay, the 2189-bp fragment from the upstream of the start codon was amplified using *OsNAC103*-specific primers (Additional file 2: Table S1). The purified DNA fragment was inserted into the vector pCAMBIA1391Z in-frame with the *GUS* reporter gene to obtain the resultant vector pCAMBIA1391Z-OsNAC103::GUS. The resultant vector was introduced into Zhonghua11 to produce transgenic plants. Tissues and organs of positive transgenic plants were collected for spatio-temporal expression pattern analysis using GUS staining assay as described by Luan et al. (2011). For GUS staining at the dark treatment, transgenic seedlings grown five days under continuous light and dark conditions, respectively, were used to perform GUS staining assay.

For the *OsNAC103* mRNA levels of different organs, RNA was isolated from SAM, root, stem, leaf and different panicles including 1 cm, 3 cm, 7 cm, 10 cm and 25 cm young panicle respectively using Trizol solution. For the *OsNAC103* mRNA levels of different developmental stages, leaves from the different stages including 30 d, 40 d, 55 d, 80 d, 90 d, 100 d, 115 d and 150 d were collected to isolate RNAs respectively using Trizol solution. Then, qRT-PCR was performed to analyze *OsNAC103* mRNA levels.

For detection of *OsNAC103* mRNA levels under various phytohormones and salt stress, one-month-old hydroponic seedlings were subjected to 100 μM ABA, MeJA, ACC by spraying or transfer to 200 mM NaCl solution to grow, respectively. For detection of *OsNAC103* mRNA levels under drought stress, one-month-old plants were subjected to drought treatment (stopping water supply). Leaf tissues were harvested at indicated times to extract RNAs. Then, qRT-PCR was performed to analyze *OsNAC103* mRNA levels.

### The Subcellular Localization of OsNAC103

The full coding sequence (CDS) without a stop codon and different truncated sequence of *OsNAC103* were amplified from 40-day-old seedling cDNA by RT-PCR using gene-specific primers (Additional file 2: Table S1). The amplified fragments were inserted into a pCAMBIA35S-GFP empty vector and fused in-frame with the green fluorescent protein (GFP) respectively to produce the resultant vectors p35S::OsNAC103-GFP, p35S::OsNAC103N1-139-GFP, p35S::OsNAC103M134-229-GFP and p35S::OsNAC103C230-346-GFP. After confirmation by sequencing, the fused vectors and empty vector were transiently transformed into tobacco epidermal cells to observe the transient expression using a laser confocal microscope (Nikon EZ-C1 Si laser confocal microscope, Japan).

### Yeast Transcriptional Activation Assay

The full-length CDS and different truncated sequences of *OsNAC103* were amplified from 40-day-old seedling cDNA by RT-PCR using gene-specific primers (Additional file 2: Table S1). *GAL4* DNA activation sequence was amplified from pGADT7 vector using *GAL4*-specific primers. The amplified sequences were respectively inserted into pGBKT7 empty vector to produce the resultant vectors pGBKT7-OsNAC103FL, pGBKT7-OsNAC103N, pGBKT7-OsNAC103C and pGBKT7-GAL4AD (positive control) and the pGBKT7 empty vector was used as a negative control. After confirmation by sequencing, the fused vectors were transformed into the yeast strain Y2H gold and the transcriptional activation was evaluated according to the growth of transformants on synthetic dropout (SD) medium SD/-Trp and SD/-Trp/-His/-Ade, respectively.

### Phytohormones, Abiotic Stresses and Darkness-Induced Leaf Senescence

*OsNAC103*-OE lines, *osnac103* mutants and WT plants were grown under hydroponic solution. For dark treatment, three-week-old plants were transferred to continuous dark condition to grow. After seven days, the phenotype was observed and Chl contents were measured, respectively. For detached leaves under dark treatment, two-month-old plant leaves were immersed in MES buffer (pH5.8) and placed in darkness for five days, then the phenotype was observed. For drought stress treatment, two-month-old plants were treated with nutrient solution containing 20% PEG6000 and the phenotype was observed and recorded after three weeks, and one-month-old seedlings grown in soil were stopped water supplement and the phenotype was observed and recorded after ten days. For ABA treatment, two-week-old seedlings were grown in hydroponic solution containing 100 μM ABA. After two weeks, the phenotype was observed and recorded. For MeJA treatment, one-month-old seedings were grown in hydroponic solution containing 100 μM MeJA. After three weeks, the phenotype was observed and recorded.

### Measurement of Chlorophyll (Chl) Contents

Chl contents were measured using the method described by Luan et al. (2011). Briefly, 0.1 g leaves were grinded and Chl was extracted using 80% acetone in the dark. The absorbance of the extract was measured using a spectrophotometer at A_645_ and A_663_. Three biological replicates were performed.

### Analysis of Shoot Gravitropic Response

Rice shoot gravitropic response was measured using five-day-old seedlings planted in plates containing 7.5% agar medium in the dark. Dehusked seeds were sterilized with 75% ethanol for 2 min and 4.5% bleach for 30 min. Then seeds were washed five times with sterilized ddH_2_O. The seedlings were then grown at 28 °C. After five days, shoot gravitropic response was investigated by measuring the shoot curvature after seedlings were rotated 90° at every 12 h interval.

### Yeast one Hybrid Assay

For the effector constructs, the full-length CDS and the truncated region (1–179 aa) of OsNAC103 were amplified by RT-PCR using gene-specific primers (Additional file 2: Table S1) and inserted into pGADT7 empty vector to produce the resultant vectors pGADT7-OsNAC103FL and pGADT7-OsNAC103N1-179. For the reporter constructs, the promoters with approximate 2 kb size from different genes (including *OsSGR*, *OsNYC1*, *OsNYC3*, *OsRCCR*, *OsPAO* and *OsSAG12*) were amplified and inserted into pLacZi vector to produce the resultant vectors pLacZi-proOsSGR, pLacZi-proOsNYC1, pLacZi-proOsNYC3, pLacZi-proOsRCCR, pLacZi-proOsPAO and pLacZi-proOsSAG12. The effector and reporter constructs were transformed into the yeast strain YM4271. The transformants were firstly grown and selected on the SD/-Ura/-Leu medium, and the positive colonies were then transferred to SD/-Ura/-Leu medium containing 5-Bromo-4-chloro-3-indolyl β-D-galactopyranoside (X-Gal). The co-transformation of OsFTL12 effector and OsMADS14 reporter were used as positive control (Zheng et al. 2023).

### Supplementary Information

**Additional file 1. Fig. S1**: Phylogenic analysis and sequence analysis of *OsNAC103*. **A** An unrooted phylogenetic tree of stress-responsive NAC (SNAC) proteins in rice and *Arabidopsis*. SNAC-A and -B are two subgroups of SNAC proteins. The tree was drawn using the Neighbor-Joining method in the MEGA 11.0 program. **B** Multiple sequence alignments between OsNAC103 and other members of the NAC subfamily in rice. (a)–(e) represent five highly conservative regions. **Fig. S2**: Phenotype and overexpression levels of *OsNAC103*-OE lines in T_0_ generation. **A** Phenotypes of *OsNAC103*-OE lines. **B** Overexpression levels of *OsNAC103*-OE lines using qRT-PCR. OE1, OE2, OE6, OE7 and OE9 are five independent *OsNAC103*-OE lines. Asterisks indicate statistically significant differences by Student’s t test (*, *P* < 0.05; **, *P* < 0.01). **Fig. S3**: Sequencing analysis of *osnac103* mutants (CR2 and CR5) by CRISPR-Cas9 system. **A** Target sites of CRISPR-Cas9 for *OsNAC103*. Solid boxes, exons; hollow box, 5′-UTR; hollow pentagon, 3′-UTR; the lines, introns; Target1, Target2 and Target3 represent three targets of *OsNAC103*, respectively. **B**–**G** Mutation sites of CR2 and CR5. Red boxes mean the position of mutations.—means deletion. Red underlines mean the position of PAM. **Fig. S4**: OsNAC103 positively regulates leaf senescence in rice. **A**–**C** Phenotype of *OsNAC103*-OE lines and *osnac103* mutants during the vegetative growth stage. Bars = 20 cm. B indicates the magnified figure in A. (C) Phenotype of *OsNAC103*-OE lines and *osnac103* mutants in the field. **D**, **E** Phenotype and total chlorophyll contents of different leaves in *OsNAC103*-OE lines and *osnac103* mutants. Leaf-2, Leaf-3, Leaf-4, Leaf-5 represent the second, third, fourth and fifth leaves of the rice plant from top down, respectively. Bar = 5 cm. OE2 and OE7 are two independent *OsNAC103*-OE lines. CR2 and CR5 are two allelic mutants. Values are shown as means ± SD, n = 3. Asterisks indicate statistically significant differences by Student’s t test (*, *P* < 0.05; **, *P* < 0.01). **Fig. S5**: Agronomic traits of *OsNAC103*-OE lines and *osnac103* mutants. **A** Panicle length, number of primary branches and 100 grains weight of *OsNAC103*-OE lines and *osnac103* mutants. Values are shown as means ± SD, n ≥ 10 individual plants. Asterisks indicate statistically significant differences by Student’s t test (*, *P* < 0.05; **, *P* < 0.01). **Fig. S6**: Expression analysis of genes associated with tiller angle and shoot gravitropism in *OsNAC103*-OE lines and *osnac103* mutants. RNAs were extracted from penultimate leaves of WT, OE lines and *osnac103* mutants at vegetative stage, and qRT-PCR was performed to analyze the relative expression levels of different genes. OE2 and OE7 are two independent *OsNAC103*-OE lines. CR2 and CR5 are two allelic mutants. Asterisks indicate statistically significant differences by Student’s t test (*, *P* < 0.05; **, *P* < 0.01). **Fig. S7**: Interaction of OsNAC103 and the promoters of leaf senescence-associated genes. Yeast cells transformed with the indicated plasmids were grown on selective SD/-Ura/-Leu medium added with X-Gal. The interaction of pLacZi-proOsMADS14 and OsFTL12 effector was as a positive control. **Fig. S8**: Alternative splices of *OsNAC103* and the blast of their amino acid sequences. **A** Three alternative splicing forms of *OsNAC103* indicated as Loc_Os07g48450.1, Loc_Os07g48450.2 and Loc_Os07g48450.3, respectively. Solid boxes, exons; hollow box, 5′-UTR; hollow pentagon, 3′-UTR; the lines, introns. **B** Alignment of amino acids sequence for the Loc_Os07g48450.1, Loc_Os07g48450.2 and Loc_Os07g48450.3. **C** RT-PCR analysis of different transcripts of Loc_Os07g48450. cDNA was obtained from leaves at ripening stage by the reverse transcription reaction. RT-PCR were performed to analyze the possible transcripts (indicated by red arrows). F, R and R’ were different primers indicated in (A). M1 and M2 were DNA ladders

**Additional file 2. Table S1: Primers used in this study.**

## Data Availability

All data generated or analysed during this study are included in this published article and its supplementary information files.

## Abbreviations

TF: Transcription factor
SAGs: Senescence-associated genes
Chl: Chlorophyll
AA: Amino acids
TRD: Transcription regulatory domain
ROS: Reactive oxygen species
NLS: Nuclear localization signal
AD: Activation domain
BD: Binding domain
SAM: Shoot apical meristem
ABA: Abscisic acid
MeJA: Methyl jasmonate
ACC: 1-Aminocyclopropane-1-carboxylic acid
DIS: Dark-induced senescence
DDI: Day of dark incubation
CDS: Coding sequence
SD: Synthetic dropout
