# Supplementary material for: OsNAC103, a NAC Transcription Factor, Positively Regulates Leaf Senescence and Plant Architecture in Rice
Source: Rice (N Y). 2024 Feb 15;17:15. doi: 10.1186/s12284-024-00690-3 (PMC10869678; doi:10.1186/s12284-024-00690-3)
Supplement: Supplementary file 1 — Additional file 1. Fig. S1: Phylogenic analysis and sequence analysis of OsNAC103. A An unrooted phylogenetic tree of stress-responsive NAC (SNAC) proteins in rice and Arabidopsis. SNAC-A and -B are two subgroups of SNAC proteins. The tree was drawn using the Neighbor-Joining method in the MEGA 11.0 program. B Multiple sequence alignments between OsNAC103 and other members of the NAC subfamily in rice. (a)–(e) represent five highly conservative regions. Fig. S2: Phenotype and overexpression levels of OsNAC103-OE lines in T0 generation. A Phenotypes of OsNAC103-OE lines. B Overexpression levels of OsNAC103-OE lines using qRT-PCR. OE1, OE2, OE6, OE7 and OE9 are five independent OsNAC103-OE lines. Asterisks indicate statistically significant differences by Student’s t test (*, P < 0.05; **, P < 0.01). Fig. S3: Sequencing analysis of osnac103 mutants (CR2 and CR5) by CRISPR-Cas9 system. A Target sites of CRISPR-Cas9 for OsNAC103. Solid boxes, exons; hollow box, 5′-UTR; hollow pentagon, 3′-UTR; the lines, introns; Target1, Target2 and Target3 represent three targets of OsNAC103, respectively. B–G Mutation sites of CR2 and CR5. Red boxes mean the position of mutations.—means deletion. Red underlines mean the position of PAM. Fig. S4: OsNAC103 positively regulates leaf senescence in rice. A–C Phenotype of OsNAC103-OE lines and osnac103 mutants during the vegetative growth stage. Bars = 20 cm. B indicates the magnified figure in A. (C) Phenotype of OsNAC103-OE lines and osnac103 mutants in the field. D, E Phenotype and total chlorophyll contents of different leaves in OsNAC103-OE lines and osnac103 mutants. Leaf-2, Leaf-3, Leaf-4, Leaf-5 represent the second, third, fourth and fifth leaves of the rice plant from top down, respectively. Bar = 5 cm. OE2 and OE7 are two independent OsNAC103-OE lines. CR2 and CR5 are two allelic mutants. Values are shown as means ± SD, n = 3. Asterisks indicate statistically significant differences by Student’s t test (*, P < 0.05; **, P < 0.01 [file 12284_2024_690_MOESM1_ESM.pptx]

## Slide 1
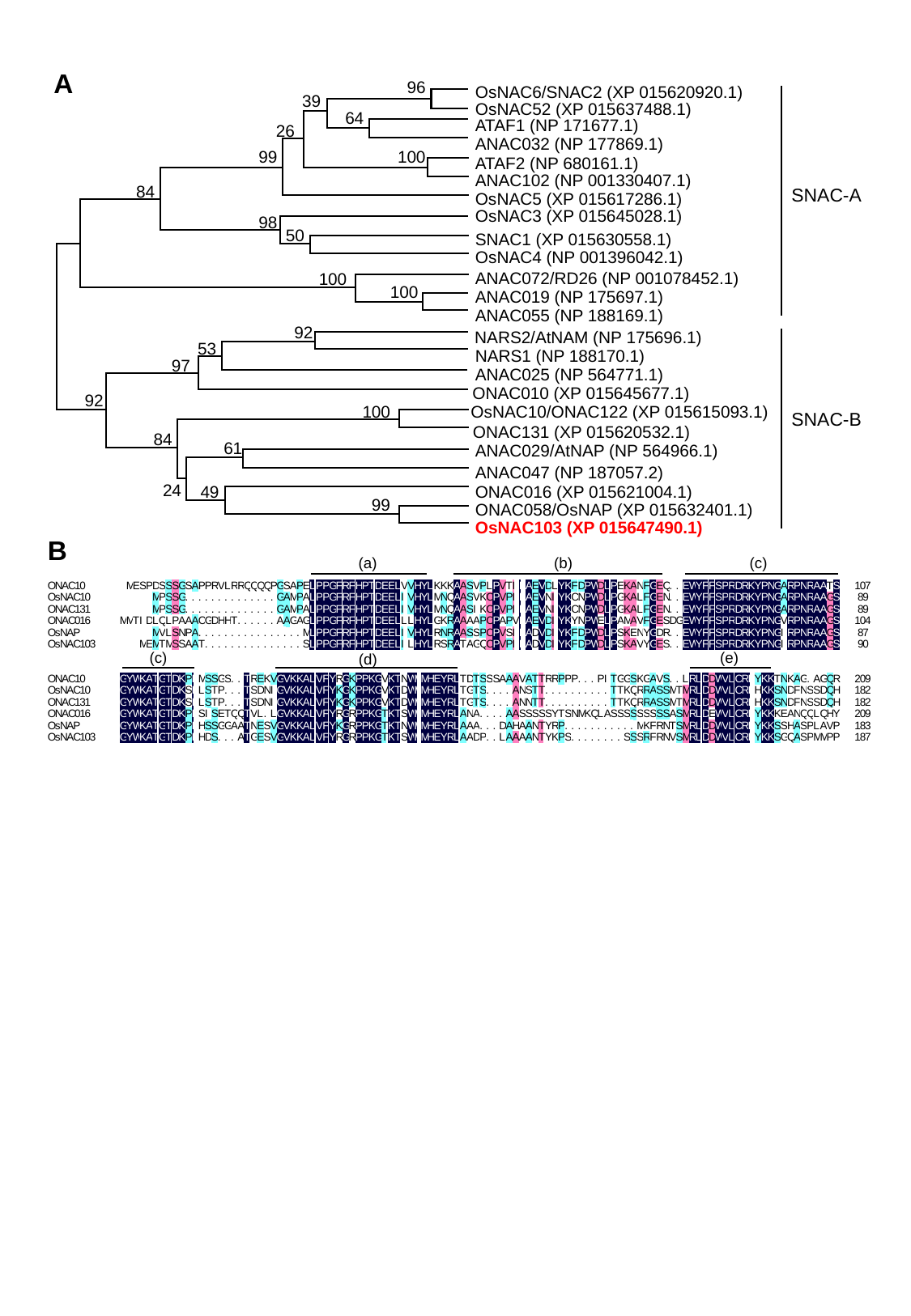

A
96
 OsNAC6/SNAC2 (XP 015620920.1)
39
 OsNAC52 (XP 015637488.1)
64
 ATAF1 (NP 171677.1)
26
 ANAC032 (NP 177869.1)
99
100
 ATAF2 (NP 680161.1)
 ANAC102 (NP 001330407.1)
SNAC-A
84
 OsNAC5 (XP 015617286.1)
 OsNAC3 (XP 015645028.1)
98
50
 SNAC1 (XP 015630558.1)
 OsNAC4 (NP 001396042.1)
 ANAC072/RD26 (NP 001078452.1)
100
100
 ANAC019 (NP 175697.1)
 ANAC055 (NP 188169.1)
92
 NARS2/AtNAM (NP 175696.1)
53
 NARS1 (NP 188170.1)
97
 ANAC025 (NP 564771.1)
 ONAC010 (XP 015645677.1)
92
100
 OsNAC10/ONAC122 (XP 015615093.1)
SNAC-B
 ONAC131 (XP 015620532.1)
84
61
 ANAC029/AtNAP (NP 564966.1)
 ANAC047 (NP 187057.2)
24
 ONAC016 (XP 015621004.1)
49
99
 ONAC058/OsNAP (XP 015632401.1)
 OsNAC103 (XP 015647490.1)
B
(a)
(b)
(c)
(c)
(e)
(d)

## Slide 2
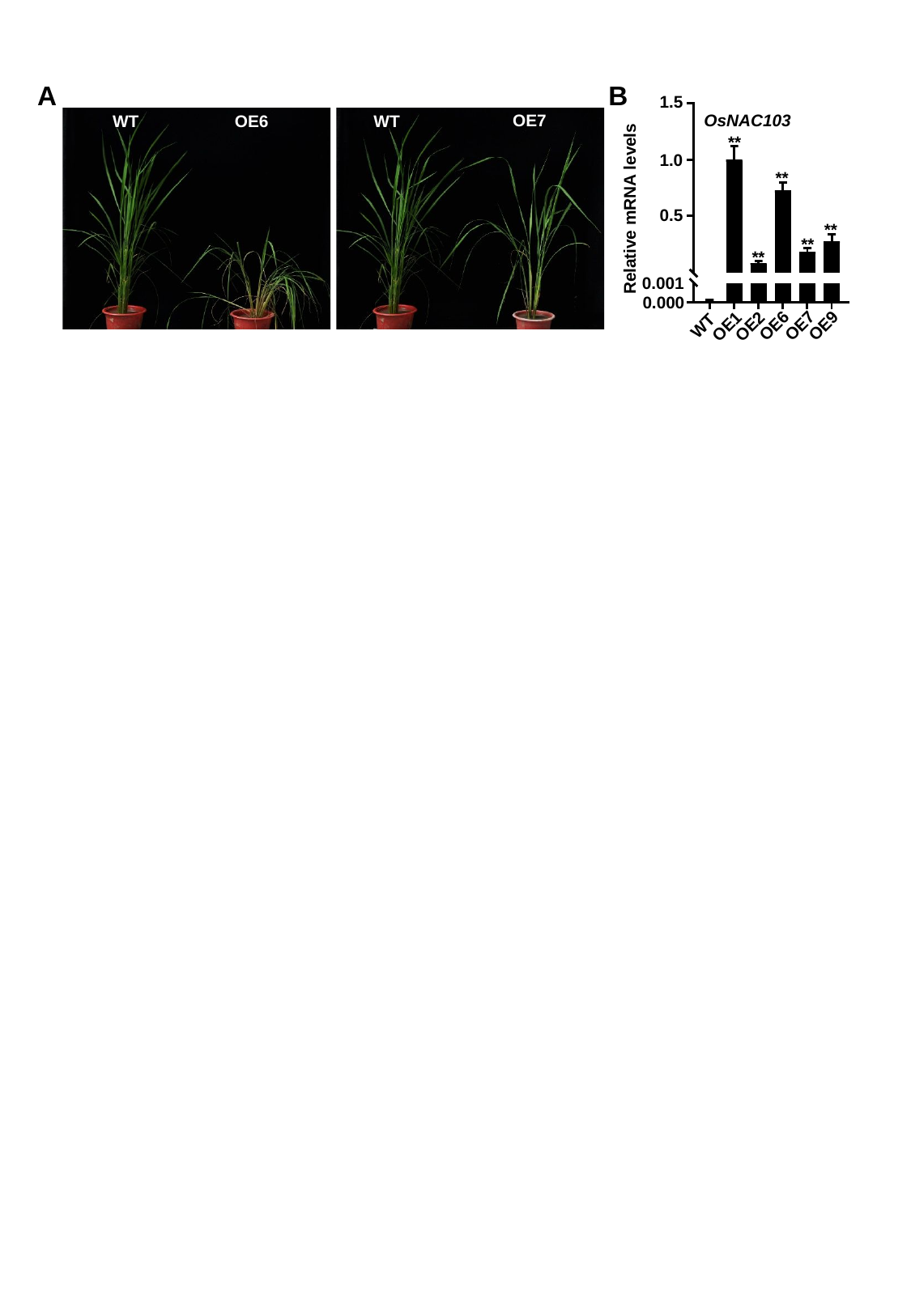

A
OE7
WT
OE6
WT
B
1.5
OsNAC103
**
1.0
**
Relative mRNA levels
0.5
**
**
**
0.001
0.000
WT
OE7
OE9
OE6
OE1
OE2

## Slide 3
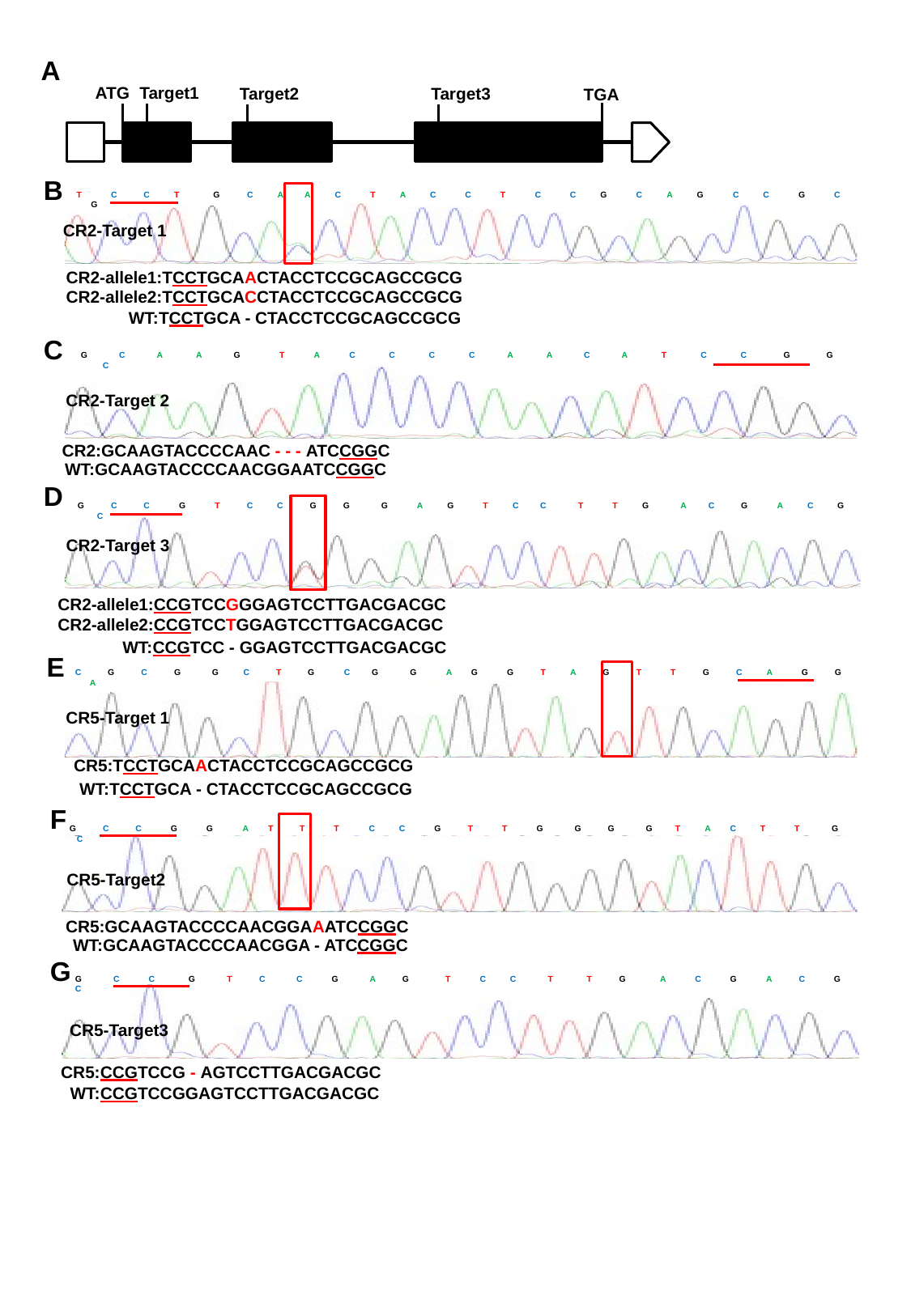

A
Target1
ATG
Target2
Target3
TGA
B
T C C T G C A A C T A C C T C C G C A G C C G C G
CR2-Target 1
CR2-allele1:TCCTGCAACTACCTCCGCAGCCGCG
CR2-allele2:TCCTGCACCTACCTCCGCAGCCGCG
WT:TCCTGCA - CTACCTCCGCAGCCGCG
C
G C A A G T A C C C C A A C A T C C G G C
CR2-Target 2
CR2:GCAAGTACCCCAAC - - - ATCCGGC
WT:GCAAGTACCCCAACGGAATCCGGC
D
G C C G T C C G G G A G T C C T T G A C G A C G C
CR2-Target 3
CR2-allele1:CCGTCCGGGAGTCCTTGACGACGC
CR2-allele2:CCGTCCTGGAGTCCTTGACGACGC
WT:CCGTCC - GGAGTCCTTGACGACGC
E
 C G C G G C T G C G G A G G T A G T T G C A G G A
CR5-Target 1
CR5:TCCTGCAACTACCTCCGCAGCCGCG
F
G C C G G A T T T C C G T T G G G G T A C T T G C
CR5-Target2
CR5:GCAAGTACCCCAACGGAAATCCGGC
WT:GCAAGTACCCCAACGGA - ATCCGGC
G
G C C G T C C G A G T C C T T G A C G A C G C
CR5-Target3
CR5:CCGTCCG - AGTCCTTGACGACGC
WT:CCGTCCGGAGTCCTTGACGACGC
WT:TCCTGCA - CTACCTCCGCAGCCGCG

## Slide 4
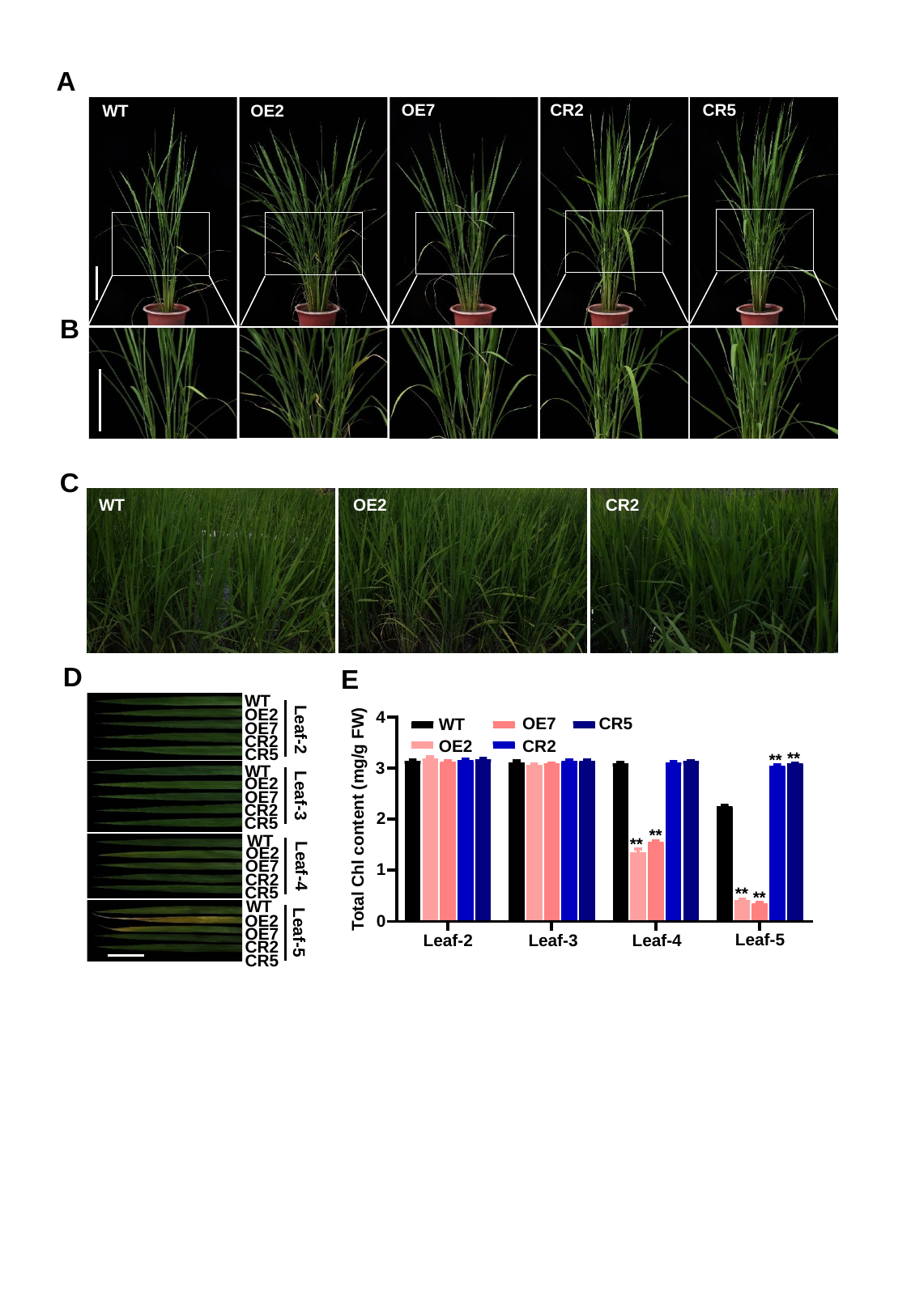

A
CR5
OE7
CR2
OE2
WT
B
C
OE2
CR2
WT
D
WT
OE2
OE7
CR2
CR5
Leaf-2
WT
OE2
OE7
CR2
Leaf-3
CR5
WT
OE2
OE7
CR2
Leaf-4
CR5
WT
OE2
OE7
Leaf-5
CR2
CR5
E
4
OE7
CR5
WT
CR2
OE2
**
**
3
Total Chl content (mg/g FW)
2
**
**
1
**
**
0
Leaf-5
Leaf-2
Leaf-3
Leaf-4

## Slide 5
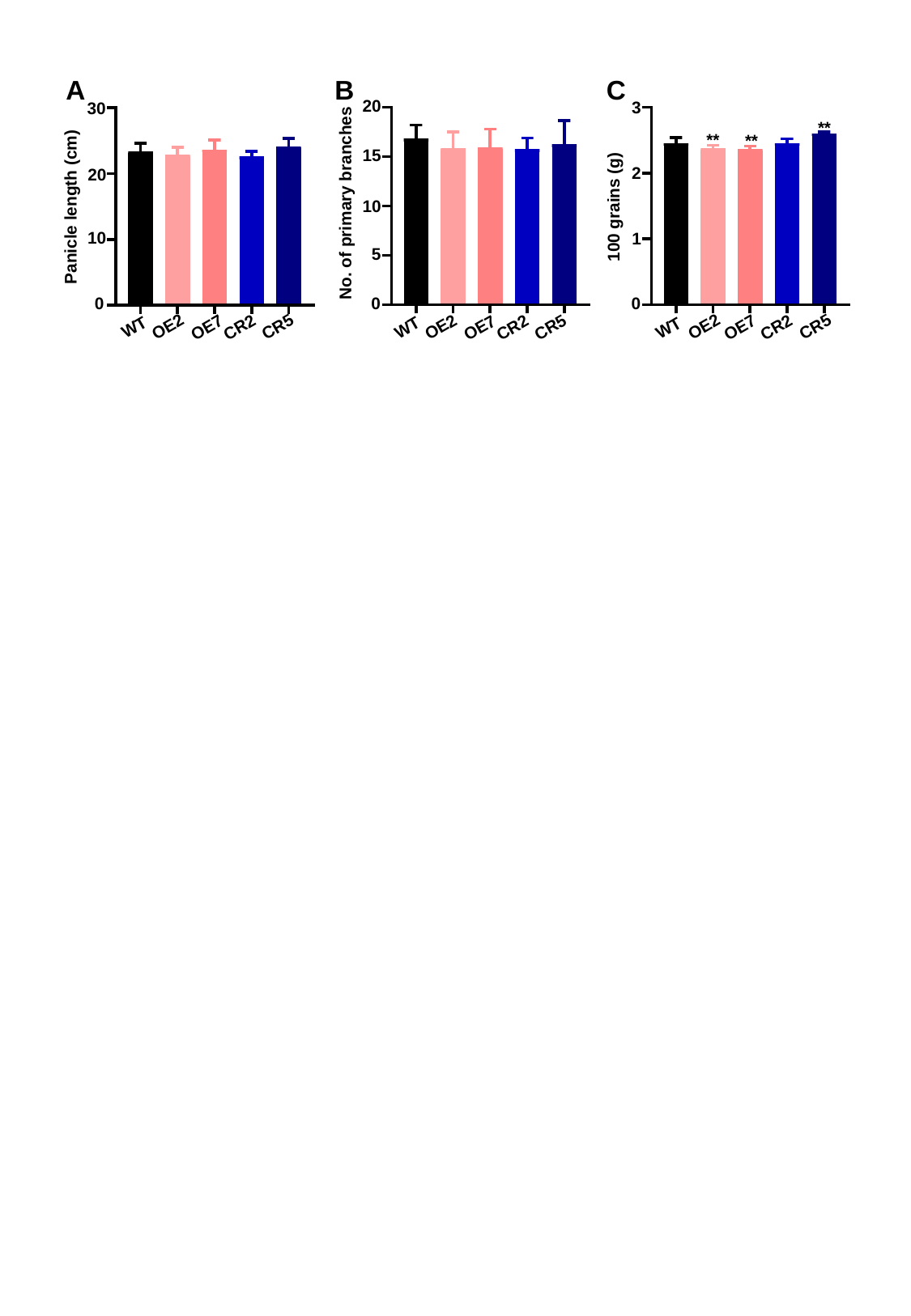

B
20
15
No. of primary branches
10
5
0
WT
OE2
CR5
CR2
OE7
C
3
**
**
**
2
100 grains (g)
1
0
OE7
CR5
OE2
CR2
WT
A
30
20
Panicle length (cm)
10
0
WT
OE2
CR5
CR2
OE7

## Slide 6
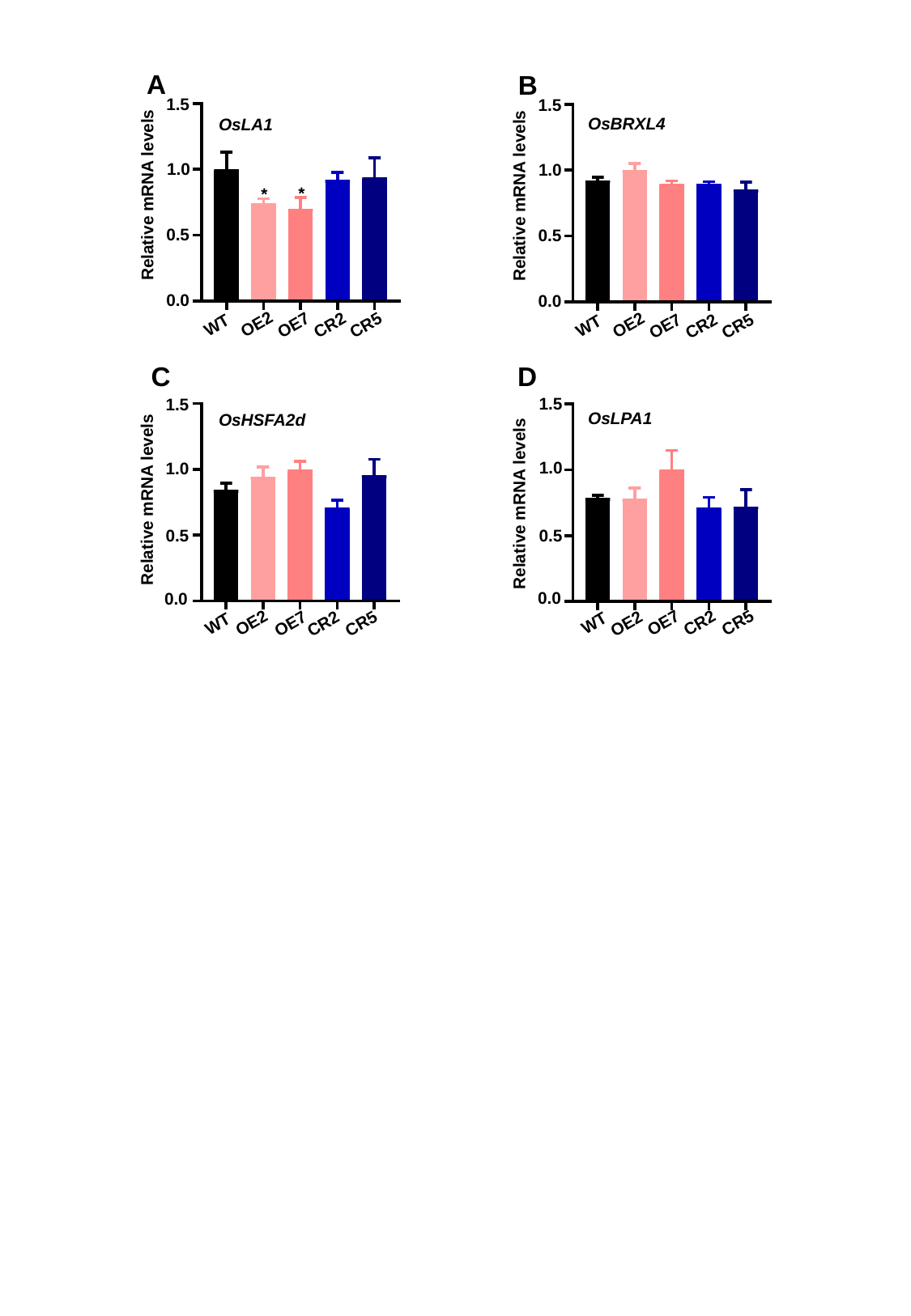

A
1.5
OsLA1
1.0
Relative mRNA levels
*
*
0.5
0.0
WT
OE2
CR5
OE7
CR2
B
1.5
OsBRXL4
1.0
Relative mRNA levels
0.5
0.0
WT
OE2
CR5
OE7
CR2
D
1.5
OsLPA1
1.0
Relative mRNA levels
0.5
0.0
WT
OE7
CR5
CR2
OE2
C
1.5
OsHSFA2d
1.0
Relative mRNA levels
0.5
0.0
WT
OE2
CR5
CR2
OE7

## Slide 7
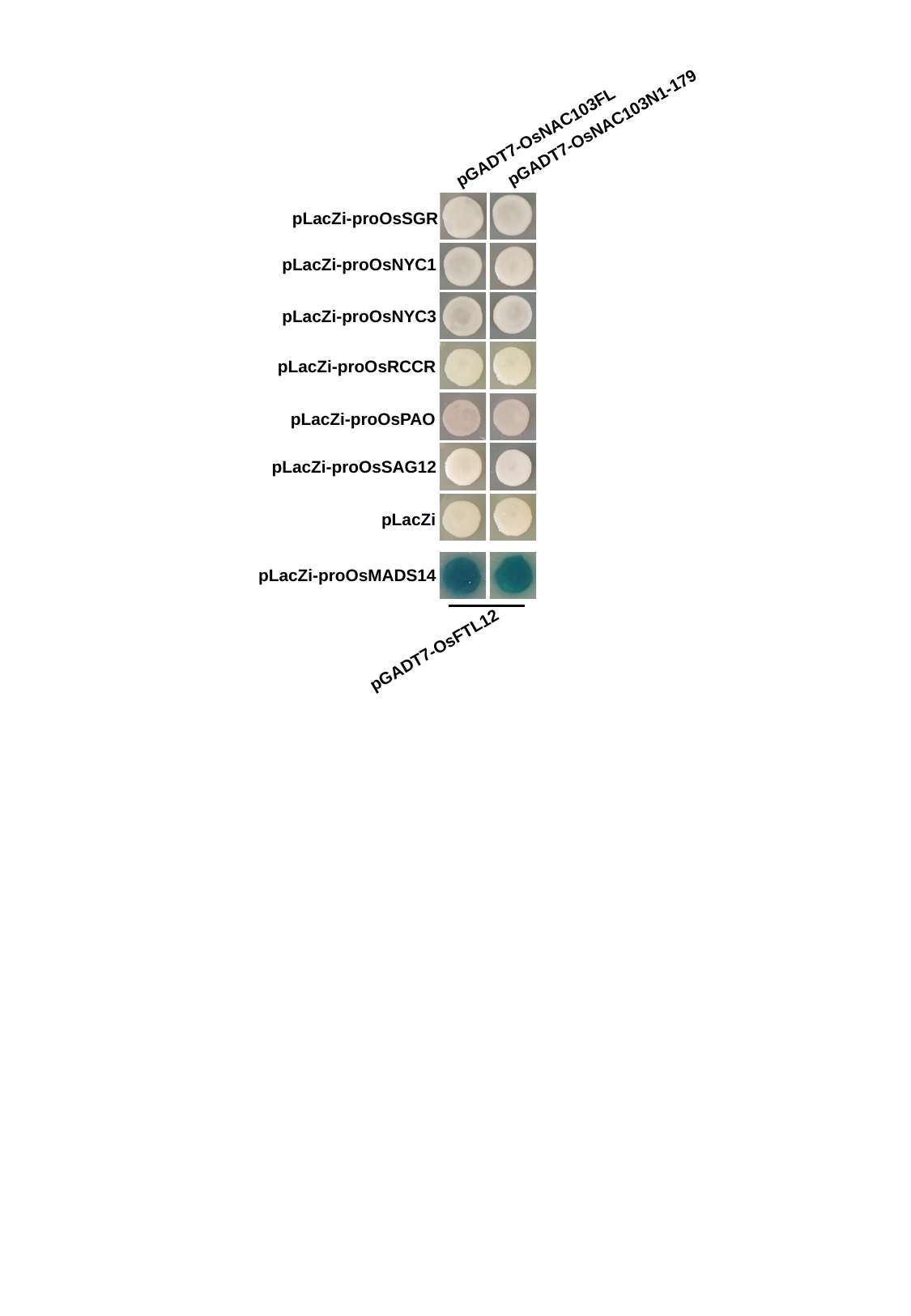

pGADT7-OsNAC103N1-179
pGADT7-OsNAC103FL
pLacZi-proOsSGR
pLacZi-proOsNYC1
pLacZi-proOsNYC3
pLacZi-proOsRCCR
pLacZi-proOsPAO
pLacZi-proOsSAG12
pLacZi
pLacZi-proOsMADS14
pGADT7-OsFTL12

## Slide 8
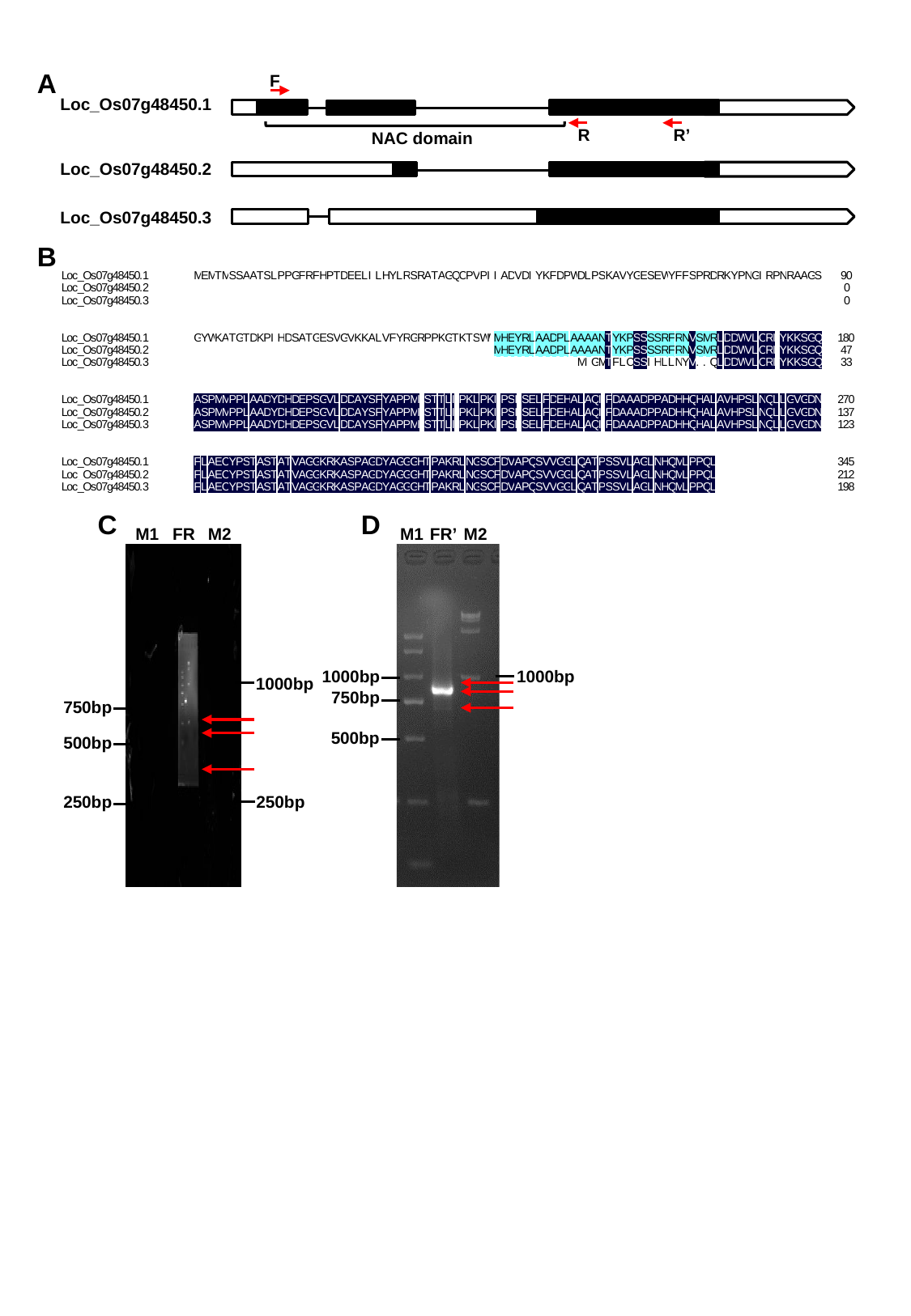

A
F
Loc_Os07g48450.1
R
R’
NAC domain
Loc_Os07g48450.2
Loc_Os07g48450.3
B
D
FR’
M2
M1
1000bp
1000bp
750bp
500bp
C
FR
M1
M2
1000bp
750bp
500bp
250bp
250bp
