# Supplementary material for: OsNAC103, a NAC Transcription Factor, Positively Regulates Leaf Senescence and Plant Architecture in Rice
Source: Rice (N Y). 2024 Feb 15;17:15. doi: 10.1186/s12284-024-00690-3 (PMC10869678; doi:10.1186/s12284-024-00690-3)
Supplement: Supplementary file 2 — Additional file 2. Table S1: Primers used in this study. [file 12284_2024_690_MOESM2_ESM.docx]

**Table S1. Primers used in this study**

| Primer name | Sequence (5’ to 3’) |
| --- | --- |
| Primers used for overexpression construction | |
| OsNAC103-OEF  OsNAC103-OER | atttggagaggacagggtaccATGGAGATGACGATGTCGTCG taaagcagggcatgcctgcagTCAGAATAGCTGAGGAGGAAGCA |
| Primers used for CRISPR/Cas9 construction and detection | |
| Target1F  Target1R  Target2F  Target2R  Target3F  Target3R  Target1Target2-TestF  Target1Target2-TestR  Target3-TestF  Target3-TestR | CGCGGCTGCGGAGGTAGTGCgttttagagctagaaat GCACTACCTCCGCAGCCGCGCggcagccaagccagca  CAAGTACCCCAACGGAATCgttttagagctagaaat  GATTCCGTTGGGGTACTTGCaacacaagcggcagc  CGTCGTCAAGGACTCCGGAgttttagagctagaaat  TCCGGAGTCCTTGACGACGCtgagcctcagcgcag  ATGGAGATGACGATGTCGTCG  CTTCTTGACGCCGACGCTCT  ATGATAGGCATGACATTTTTGTGC  TCAGAATAGCTGAGGAGGAAGCA |
| Primers used for qRT-PCR analysis | |
| OsNAC103F  OsNAC103R  OsSGRF  OsSGRR  OsNOLF  OsNOLR  OsNYC1F  OsNYC1R  OsNYC3F  OsNYC3R  OsPAOF  OsPAOR  OsRCCRF  OsRCCRR  OsSAG12F  OsSAG12R  OsNAPF  OsNAPR  OsABA8ox3F  OsABA8ox3R  OsABI5F  OsABI5R  OsbZIP23F  OsbZIP23R  OsLOX4F  OsLOX4R  OsAOS2F  OsAOS2R  OsAOCF  OsAOCR  OsOPR7F  OsOPR7R  OsCOI1bF  OsCOI1bR  OsLPA1F  OsLPA1R  OsHSFA2dF  OsHSFA2dR  OsBRXL4F  OsBRXL4R  OsbZIP49F  OsbZIP49R  OsLA1F  OsLA1R  OsPIN1F  OsPIN1R  OsPIN2F  OsPIN2R  OsActin1F  OsActin1R | ATCCCCAAGCTCCCCAAGAT  AAGCATGAGCCGTTGAGCCT  ACGAGGAGAAGCACCAGCAC  GCGGAAGATGTAGTAGCGGA  TCGATGGTGCTGGTTCTGAT  CCGCAACCACATTAGCAGGT  TTCCGAGGATGCGAGTTGTG  ACGACAGAGAGCGAGAACAC  GGCGGAGGAGTTGGTGTATT  AAGCGAGGAGATGTAGCAGG  CGAGAATGGGTGGGAGAAGG  CAGTGACCTTGTGGTGAGCA  CTGCAAAGTCCCAAATGGCG  CGCTGTTTGTCCACCTGAGT  CTGGCTGATGAAGAACTCGT  AGAAACAGAGCACCATCCAT  GGAGAATTACGGGGACAGGG  AGTTGGTCTTGGTGCCCTTG  CTTACATCGGCGAGACCCTT  CGATCATCCGCTCCTTGCTC  ATGGTTGTCGTGAGTCCTGG  AGCTCCACCGTATAAGCCTG  TGTGTTCCCTCCGATGGTGC  CTTCCCCTCAGCCCACCTTT  TGAGCGAGCCTATGATCCAG  ATGGTCATCGTGTCGCGGTA  AAGAAGGGGGAGATGCTGTT  AACGCTAGGGTTCTCCGTCT  CTGCCTCAACAACTTCACCAACT  CGCACATGCCGCAATTAACAC  GCTCCTATGGCAAGTATCCT  ACCACAGCCCTAGTTACCTC  GGTACAGGGCTACAAGGCTT  GGCAAGAGGCAAGAACGAAG  ACCTCGTCGGCATCAAGAAG  ATCGTCGTCGCGTGTTGCTC  GCTCCGATTTGTCCAACCAT  GGCTCAACTTTACATCACCAGA  CAGCAAACTCCCCTTCCTAC  CTTAGGTGTTGATGCCAGCC  CTAGGCTGGTTGGTGCTGAC  AGGTTGCTGTAGCTGCCGTA  AAAGGCGGCGGCTACAAGAC  CGCTCTTGTTGCCGTTCATC  AACCCGAACACCTACTCCAG  GCCATGAACAGACCGAGACT  CATCAGAAACCCCAACACCT  ACGGTCTTGCCACAAGAAAT  GACTCTGGTGATGGTGTCAGC  GGCTGGAAGAGGACCTCAGG |
| Primers used for GUS staining assay | |
| OsNAC103-GUSF  OsNAC103-GUSR | gaccatgattacgccaagcttCCTTCTACAGTGGCTCTCCTATGAC  ccagtgaattcccggggatccCGACGATCTAATACGGTTACTGCT |
| Primers used for subcellular localization | |
| OsNAC103-GFPF  OsNAC103-GFPR  N1-139-GFPF  N1-139-GFPR  M134-229-GFPF  M134-229-GFPR  N230-346-GFPF  N230-346-GFPR | acgggggacgagctcggtaccATGGAGATGACGATGTCGTCG  ggtgtcgatcctagaggatccGAATAGCTGAGGAGGAAGCATCTG  acgggggacgagctcggtaccATGGAGATGACGATGTCGTCG  ggtgtcgactctagaggatccGAGGCGGTACTCGTGCATGA  acgggggacgagctcggtaccATGCACGAGTACCGCCTCG  ggtgtcgactctagaggatccCTCGGAGATGGAGGGGATCT  acgggggacgagctcggtaccATGCTCTTCGACGAGCACGC  ggtgtcgatcctagaggatccGAATAGCTGAGGAGGAAGCATCTG |
| Primers used for yeast transcriptional activation assay | |
| OsNAC103FL-BDF  OsNAC103FL-BDR  OsNAC103N-BDF  OsNAC103N-BDR  OsNAC103C-BDF  OsNAC103C-BDR  GAL4AD-BDF  GAL4AD-BDR | tcagaggaggacctgcatatgATGGAGATGACGATGTCGTCG  tcgacggatccccgggaattcTCAGAATAGCTGAGGAGGAAGCA  tcagaggaggacctgcatatgATGGAGATGACGATGTCGTCG  tcgacggatccccgggaattcGCCGGACTTCTTGTAGATCCG  tcagaggaggacctgcatatgCAGGCGTCGCCGATGATG  tcgacggatccccgggaattcTCAGAATAGCTGAGGAGGAAGCA  tcagaggaggacctgcatatgTTTAATCAAAGTGGGAATATTGCTG  tcgacggatccccgggaattcCTCTTTTTTTGGGTTTGGTGGG |
| Primers used for yeast one hybrid assay | |
| OsNAC103FL-ADF  OsNAC103FL-ADR  OsNAC103N-ADF  OsNAC103N-ADR  proOsSGR-pLacZiF  proOsSGR-pLacZiR  proOsNYC1-pLacZiF  proOsNYC1-pLacZiR  proOsNYC3-pLacZiF  proOsNYC3-pLacZiR  proOsPAO-pLacZiF  proOsPAO-pLacZiR  proOsRCCR-pLacZiF  proOsRCCR-pLacZiR  proOsSAG12-pLacZiF  proOsSAG12-pLacZiR | gtaccagattacgctcatatgATGGAGATGACGATGTCGTCG  atgcccacccgggtggaattcTCAGAATAGCTGAGGAGGAAGCA  gtaccagattacgctcatatgATGGAGATGACGATGTCGTCG  atgcccacccgggtggaattcGCCGGACTTCTTGTAGATCCG  cttgaattcgagctcggtaccAGGTGAAAGAGAGAGCGGAGAGT  atacagagcacatgcctcgagGTCTGCTCCCTCGGATCTCTTA  cttgaattcgagctcggtaccATACTCGTCTGCGGTGGCTT  atacagagcacatgcctcgagCCGCGAGCGGATAAGGATTT  cttgaattcgagctcggtaccCACCGCTCCCTGCCCATA  agcacatgcctcgaggtcgacACACTAGTGGCTAGCACGCTAGTT  tgaattgaaaagcttgaattcCACTGCCTGTTCATGTCCAC  gtcgacagatccccgggtaccGGCGTTTTGCGGGGTGTC  cttgaattcgagctcggtaccCTATTTGACAAGGGAACTGAACCC  atacagagcacatgcctcgagTGGATCACGGCGAGGTGTT  tgaattgaaaagcttgaattcAACAACGTTAGCGGTTGTCTACC  atacagagcacatgcctcgagGTGGGTGATTGTTTAGCTTGGTT |
| Primers used for the validation of different splicing forms | |
| F  R  R’ | ATCCTGCACTACCTCCGCAG  GTAGGCGTCGTCAAGGACTC  AAGCATGAGCCGTTGAGCCT |
